# The interplay of homeostasis, inflammation, and oxidative stress in neurodegenerative disorders: the role of biological markers, antioxidants, lithium, and TMS — a proposed framework for preventing neurodegenerative disorders through biomarkers and multimodal therapies

**DOI:** 10.3389/fnagi.2025.1607669

**Published:** 2026-01-09

**Authors:** Leonard Lado, Aruna Misir

**Affiliations:** 1 Lado Healing Institute, Bonita Springs, FL, United States; 2 Xavier University School of Medicine, Oranjestad, Netherlands

**Keywords:** neurodegenarative disease, oxidative stress, biomarkers, lithium, transcranial magenetic stimulation

## Abstract

Neurodegenerative diseases such as Alzheimer’s disease (AD) and Parkinson’s disease (PD) are among the most significant health challenges of aging, characterized by progressive cognitive and motor decline. Increasing evidence suggests that these conditions are not inevitable outcomes of aging but may instead be driven by preventable mechanisms involving oxidative stress, chronic inflammation, and disruptions in homeostasis. This manuscript proposes a preventive framework that integrates validated biomarkers: glial fibrillary acidic protein (GFAP), neurofilament light chain (NfL), soluble triggering receptor expressed on myeloid cells 2 (sTREM2), and YKL-40 or CHI3L1 by its more commonly used name: Chitinase-3-like protein 1 with multimodal therapeutic interventions, including antioxidants, lithium, and transcranial magnetic stimulation (TMS). Oxidative stress is positioned as a central mediator of neurodegeneration, with biomarkers serving as early indicators that enable detection before irreversible neuronal loss. This supports our proposal that NfL is not only a marker of pathology but also a measurable indicator of lithium’s effect in stabilizing axons and reducing neurodegeneration. These results align with our framework, which places TMS as a synergistic tool with lithium and antioxidants to modify both oxidative and neuroplastic pathways with a translational preventive strategy. Importantly, recent findings published in demonstrated that reducing dietary lithium by more than 50% in AD mouse models accelerated amyloid-*β* and tau pathology, increased microglial activation, and led to cognitive decline. Remarkably, lithium supplementation prevented these changes and preserved neuronal and cognitive function. These results provide powerful preclinical validation of our framework, reinforcing the concept that lithium deficiency may be pathogenic and that restoring physiological lithium levels could serve as a preventive therapy. The model also incorporates viral contributions (HSV-1, EBV) as triggers of chronic inflammation and amyloid pathology, providing a more comprehensive view of disease initiation. It further emphasizes the potential synergy of combining antioxidants with TMS, highlighting avenues for multimodal prevention. These findings reinforce the role of inflammation as both a driver and a modifiable factor in neurodegeneration. Our model integrates lithium’s anti-inflammatory effects with biomarker monitoring (e.g., YKL-40, sTREM2) to translate these insights into targeted preventive strategies. These results align with our framework, which places TMS as a synergistic tool with lithium and antioxidants to modify both oxidative and neuroplastic pathways, bridging state-of-the-art findings with a translational preventive strategy. We acknowledge limitations, including the need for improved biomarker specificity and sensitivity, inconsistent outcomes of antioxidant trials, the accessibility and cost of TMS, and the therapeutic window of lithium. Nonetheless, by reframing AD and PD as preventable rather than inevitable, our framework highlights a proactive approach that integrates molecular mechanisms, biomarkers, and multimodal therapies into a cohesive strategy with both scientific promise and translational potential.

## Introduction

Parkinson’s disease (PD) and Alzheimer’s disease (AD) do not occur in a vacuum. These conditions result from years, often decades, of subtle imbalances accumulating within the brain. Inflammation and oxidative stress, natural defense mechanisms against threats, become destructive when unchecked (Butterfield and Halliwell, 2019). The immune system, in its attempt to heal, can inadvertently perpetuate the damage it seeks to repair (Chapleau et al., 2022).

Understanding the interplay of these processes offers a powerful message: PD and AD are not the inevitabilities they once seemed. They are diseases that we, as a scientific and medical community, can prevent. But to do so, we must think differently, embracing early detection, refining interventions, and addressing the environmental and lifestyle factors that fuel these conditions (Janigro et al., 2022). A crucial component of this framework is the implementation of reliable biological markers for early detection. Biomarkers such as malondialdehyde (MDA), 4-hydroxynonenal (4-HNE), carbonylated proteins, mitochondrial activity markers, and more recently validated markers including GFAP, NfL, sTREM2, and YKL-40, provide measurable indicators of oxidative stress, inflammation, and neuronal injury (Villa et al., 2020). These form the foundation of an early intervention strategy, enabling clinicians to disrupt pathological processes before irreversible neuronal damage occurs. The biomarker-based temporal model of Alzheimer’s pathology, popularized by Jack et al. (2010), highlights that neurodegeneration begins decades before clinical symptoms appear. By employing high-precision assays in cerebrospinal fluid (CSF), blood, or urine, at-risk individuals can be identified and preventive treatments applied long before symptoms manifest (Itzhaki et al., 2016).

**Figure:**
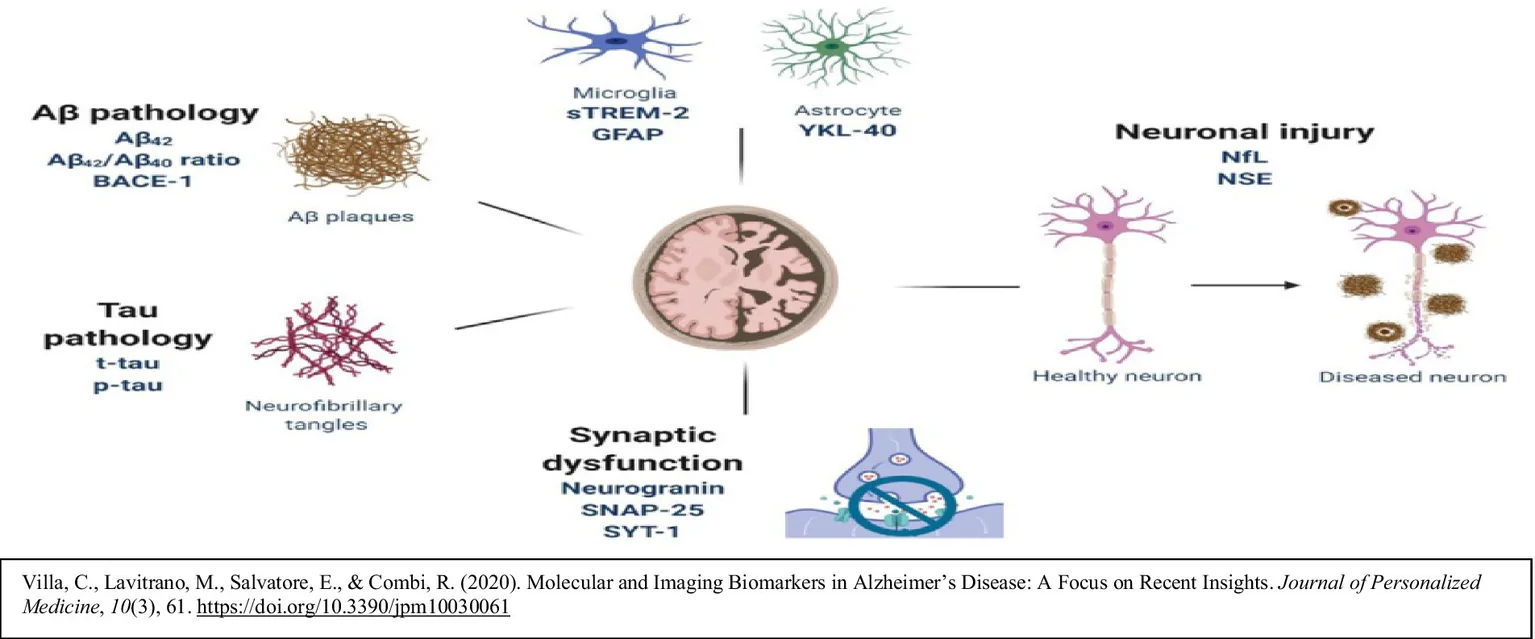


Recent advances also highlight under-recognized contributors such as viral influences. Herpes simplex virus type 1 (HSV-1) and Epstein–Barr virus (EBV) are increasingly implicated in driving chronic inflammation and amyloid aggregation, underscoring the need to view neurodegeneration in the context of persistent environmental triggers (Bjornevik et al., 2022). Similarly, nutritional and metabolic factors deserve greater attention. An often-overlooked element in neurodegenerative vulnerability is lithium status. Historically, dietary lithium intake may have declined due to changes in water and food sources (Aron et al., 2025). This concept has recently been validated in a groundbreaking *Nature* study from Harvard (2025), which showed that reducing dietary lithium by ~50% in AD mouse models accelerated amyloid-*β* and tau pathology, increased microglial activation, and led to cognitive decline, whereas lithium orotate supplementation prevented these changes and preserved neuronal function (Aron et al., 2025). These findings support lithium deficiency as a nutritional and environmental risk factor and provide a rationale for early preventive correction. Environmental studies have demonstrated that naturally occurring lithium levels in drinking water correlate with reductions in suicidality and neuropsychiatric disturbances, further supporting lithium’s biological relevance (Schrauzer and Shrestha, 1990).

This document presents a proposed framework for the development of Alzheimer’s disease (AD) and Parkinson’s disease (PD). Current evidence suggests that these diseases may be preventable and detectable at very early stages through the use of biomarkers (Butterfield and Halliwell, 2019). By identifying early markers and employing early detection strategies, it may be possible to intervene with modalities that influence oxidative processes, thereby reducing the formation of harmful radicals (Chapleau et al., 2022).

The novelty of this manuscript does not rest on re-stating oxidative stress and inflammation as mechanisms, but rather in integrating validated biomarkers, multimodal therapies (antioxidants, lithium, TMS), viral contributions, and translational/economic perspectives into a single cohesive framework. This work refines and unifies existing theories into a preventive model that emphasizes early detection, combined interventions, and opportunities for innovation in clinical and pharmaceutical development. Foundational redox biology work has demonstrated how oxidative signaling pathways drive neuronal vulnerability, a theme expanded upon by Chiu et al. (2013) showing how redox-inflammatory interactions shape neurodegenerative trajectories.

Although this paper focuses primarily on Alzheimer’s disease as a case example, references to Parkinson’s disease are included to illustrate how similar pathways of oxidative stress, inflammation, and homeostatic disruption may apply across neurodegenerative disorders. A fuller discussion of PD within this framework will be addressed in future work.

Comparison of Alzheimer’s disease (AD) and Parkinson’s disease (PD).

**Table 1:** 

Aspect	Alzheimer’s disease (AD)	Parkinson’s disease (PD)
Oxidative stress	The Alzheimer’s brain is highly vulnerable to oxidative stress due to its high oxygen consumption and lipid-rich composition. Excess reactive oxygen species (ROS) lead to lipid peroxidation, protein oxidation, and DNA damage, driving synaptic dysfunction, tau hyperphosphorylation, and amyloid-β aggregation.	Dopaminergic neurons in the substantia nigra are uniquely susceptible to oxidative stress. Dopamine metabolism itself generates ROS, and combined with mitochondrial dysfunction, this results in elevated oxidative burden, promoting progressive dopaminergic neuronal loss.
Neuroinflammation	Chronic activation of microglia and astrocytes around amyloid plaques leads to persistent release of pro-inflammatory cytokines such as IL-1β, IL-6, and TNF-*α*. This environment amplifies neuronal injury, disrupts synaptic plasticity, and accelerates neurodegeneration.	Microglial overactivation within the substantia nigra results in sustained production of inflammatory mediators including TNF-α, IL-1β, and IL-6. This creates a self-perpetuating cycle of inflammation and oxidative stress, driving dopaminergic neurodegeneration and contributing to both motor and cognitive symptoms.
Key biomarkers	Elevated glial fibrillary acidic protein (GFAP) reflects astrocytic activation and BBB disruption. Neurofilament light chain (NfL) increases with axonal damage and predicts disease progression. Soluble TREM2 (sTREM2) rises with microglial activation, YKL-40 reflects glial inflammation and tissue remodeling, and S100B indicates BBB dysfunction. Collectively, these biomarkers provide a multi-dimensional picture of neurodegeneration.	GFAP elevation is associated with astrocytic activation. NfL is elevated in dopaminergic neuronal injury and progression of motor and non-motor decline. sTREM2 indicates microglial activation in the substantia nigra, while YKL-40 and S100B reflect ongoing glial-driven inflammation and BBB dysfunction. These markers parallel those in AD, highlighting common pathways of degeneration.
Lithium’s role	Lithium inhibits GSK-3β, reducing tau hyperphosphorylation and amyloid-*β* production. It stabilizes mitochondria, reduces ROS generation, and enhances BDNF signaling supporting synaptic plasticity and neuroprotection. The Harvard Nature 2025 study provided compelling evidence that lithium deficiency accelerates Alzheimer’s pathology, while supplementation prevented decline, validating lithium as both a nutrient and therapeutic agent.	In PD, lithium improves mitochondrial function, reduces ROS, and inhibits GSK-3β. It protects dopaminergic neurons from apoptosis and modulates autophagy, enhancing clearance of damaged proteins and organelles. These effects highlight lithium’s potential as a neuroprotective and disease-modifying therapy, especially in early or prodromal stages.
Transcranial Magnetic Stimulation (TMS)	Repetitive TMS (rTMS) reduces oxidative stress biomarkers, enhances BDNF levels, and improves connectivity between parietal and hippocampal regions. Clinically, this translates into improvements in cognition, memory, and mood, underscoring its role as a non-invasive neuromodulator therapy for AD.	High frequency rTMS targeting the motor cortex has shown improvements in motor symptoms such as bradykinesia and rigidity. In addition, rTMS may enhance cognition, reduce depression, and improve gait and daily functioning in PD, making it a promising adjunctive treatment in multimodal strategies.

## Proposed theory on the development of Alzheimer’s disease and Parkinson’s disease

This document presents my proposed theory on the development of Alzheimer’s disease (AD) and Parkinson’s disease (PD). I believe that these diseases are preventable and can be detected at extremely early stages through the use of biomarkers (Butterfield and Halliwell, 2019). By identifying early markers and employing early detection strategies, we can intervene with modalities that positively impact the oxidative process, disrupting the formation of harmful radicals (Chapleau et al., 2022).

My proposed model emphasizes dual approaches, such as the use of transcranial magnetic stimulation (TMS) combined with antioxidant therapies, to address oxidative stress and mitigate its harmful effects (Janigro et al., 2022). In the case of viral contributions to neurodegeneration, my model also suggests that timely intervention can prevent the progression of viral damage in the brain (Villa et al., 2020). This vulnerability highlights why oxidative stress reduction is a cornerstone of our model. By combining lithium, antioxidants, and TMS, our framework directly targets these ROS-driven processes in a way that current therapies do not. These results align with our framework, which places TMS as a synergistic tool with lithium and antioxidants to modify both oxidative and neuroplastic pathways, bridging state-of-the-art findings with a translational preventive strategy.

What makes this theory unique is that it does not introduce entirely new concepts but rather connects existing scientific data into a cohesive and comprehensive model. The implications of this model are significant, as it proposes a standard in which AD and PD can be treated before they worsen, offering the potential for better patient outcomes (Chapleau et al., 2022).

I recognize that such a proposal may raise concerns about its impact on current treatment models for Alzheimer’s and Parkinson’s, potentially affecting large corporations economically. However, I view this shift not as a threat but as an opportunity; an opportunity to develop new compounds, technologies, and therapies with superior outcomes for patients. Every time a new treatment emerges; it brings with it a vast economic potential. This model should be seen as a pathway to innovation and growth for many companies in the field of neurodegenerative disease treatment (Janigro et al., 2022).

Additionally, my model highlights that by intervening in the oxidative process early, through strategies such as TMS and targeted antioxidant therapy, the progression of neuronal damage can be disrupted, ultimately preserving cognitive and motor functions (Villa et al., 2020).

Transcranial magnetic stimulation (TMS) is proposed as a complementary therapeutic strategy in this model because of accumulating evidence that it reduces oxidative stress, enhances neuroplasticity, and strengthens connectivity between cortical and hippocampal regions. These mechanisms directly align with the oxidative stress–centered framework presented here, supporting the rationale for TMS as one of the key preventive and therapeutic approaches (Bashir et al., 2022; Velioglu et al., 2021). This vulnerability highlights why oxidative stress reduction is a cornerstone of our model. By combining lithium, antioxidants, and TMS, our framework directly targets these ROS-driven processes in a way that current therapies do not.

Importantly, in cases involving viral infections, addressing reactivation and chronic inflammation in the brain could prevent further deterioration (Itzhaki et al., 2016). These findings reinforce the role of inflammation as both a driver and a modifiable factor in neurodegeneration. Our model integrates lithium’s anti-inflammatory effects with biomarker monitoring (e.g., YKL-40, sTREM2) to translate these insights into targeted preventive strategies.

It is crucial to note that while current treatments focus on symptomatic relief, my model emphasizes prevention and early-stage intervention. Unlike many current approaches that primarily address symptoms, my model offers a path toward a true cure by halting disease progression before it reaches advanced stages (Mao and Reddy, 2011). Furthermore, even in cases where early intervention is not possible, the proposed methods can still alleviate symptoms and improve a patients’ quality of life (Mao and Reddy, 2011). Such a shift in focus offers significant potential to improve patient outcomes while opening new avenues for pharmaceutical and technological innovations. For the sake of simplicity and to introduce a new concept in treatment, I will focus solely on Alzheimer’s disease in this publication, emphasizing my oxidative stress model. Due to space and time limitations, I will present the same theory for my model regarding Parkinson’s disease in a future paper as I believe the underlying mechanism can apply to both conditions.

## The role of homeostasis in inflammation and oxidative stress

Under normal conditions, homeostasis ensures a balance between pro-inflammatory and anti-inflammatory responses. This balance is maintained through immune cell signaling, cytokine pathways, and central nervous system regulation. Disruption of this balance, due to chronic stress, poor nutrition, or genetic predisposition, leads to a vicious cycle of oxidative stress and inflammation (Merelli et al., 2021; Biswas, 2016).

### Oxidative stress and mitochondrial dysfunction

Oxidative stress results from an imbalance between reactive oxygen species (ROS) production and antioxidant defenses. Seminal work by Beal (2003) established mitochondrial function is compromised, ROS levels increase, causing lipid peroxidation, protein oxidation, and DNA damage. These events further impair cellular functions and contribute to neurodegeneration (Beurel et al., 2010; Sbodio et al., 2019). Mitochondrial impairment is one of the earliest drivers of neuronal vulnerability, creating an energy deficit that amplifies oxidative stress and accelerates neurodegenerative mechanisms (Beal, 2003).

### Chronic inflammation and neurodegeneration

“This chronic inflammatory state is a major contributor to the progression of neurodegenerative diseases, acting in concert with oxidative stress and other pathological mechanisms” (Nathan and Ding, 2010; Biswas et al., 2016).

1 Monitoring and feedback loops

- The body constantly monitors internal conditions through immune cells, cytokine signaling pathways, and the central nervous system (Finkel, 2011).
- For instance, damaged cells release damage-associated molecular patterns (DAMPs) that signal immune cells to the site of injury (Birben et al., 2012).

2 Control of reactive oxygen species (ROS)

- Under homeostatic conditions, the production and neutralization of ROS are tightly regulated by antioxidant systems such as superoxide dismutase (SOD) and glutathione peroxidase (Serhan and Savill, 2005).
- When oxidative stress overwhelms these defenses, feedback mechanisms signal for increased antioxidant production (Medzhitov, 2008).

3 Resolution of inflammation

- The inflammatory response is self-limiting under normal conditions. Once the threat is resolved, pro-inflammatory signals are downregulated, and anti-inflammatory mediators like resolving and lipoxins restore tissue homeostasis (Itzhaki et al., 2016).
- Chronic inflammation occurs when these homeostatic mechanisms fail, often due to persistent stressors or immune dysregulation (Pickles et al., 2018).

**Figure:**
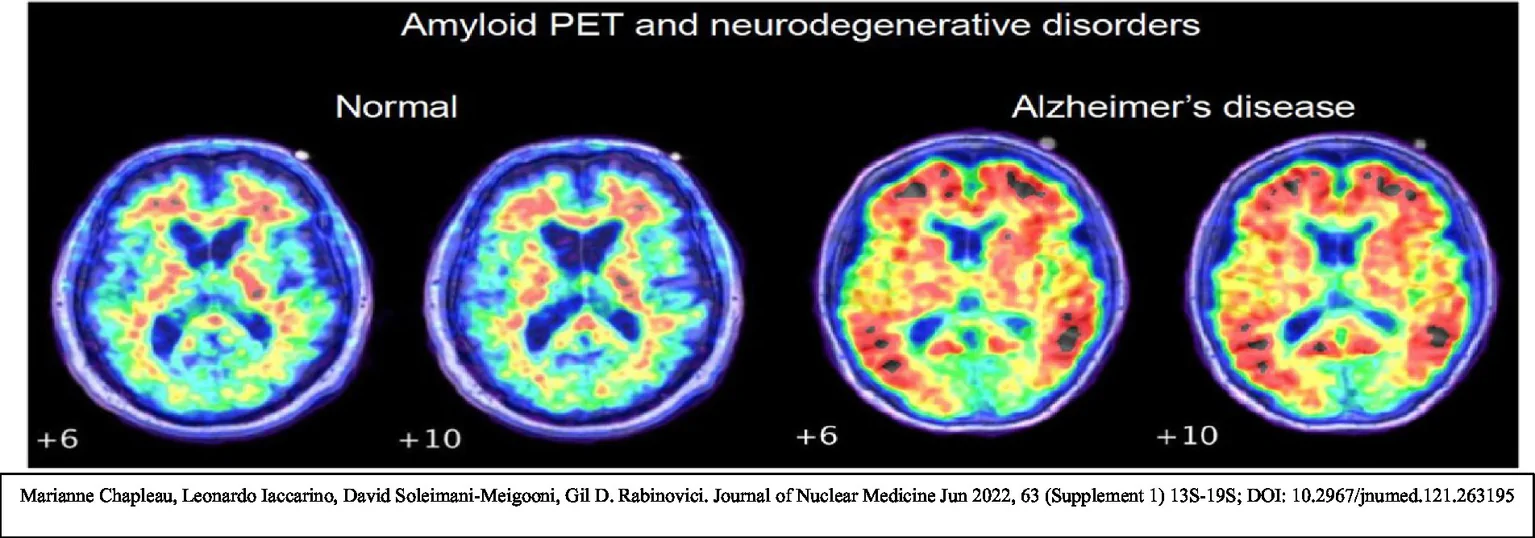


### Proposed model of oxidative stress of Alzheimer’s disease

Alzheimer’s disease is a condition that robs individuals of their memories, relationships, and ultimately their sense of self. At its core, Alzheimer’s disease is characterized by the accumulation of amyloid-beta plaques and hyperphosphorylated tau tangles, which disrupt neural communication and lead to cell death. However, these visible hallmarks are only part of the story.

1. *Oxidative stress and inflammation in Alzheimer’s disease*: The brain’s high oxygen consumption and lipid-rich environment make it particularly susceptible to oxidative damage. In Alzheimer’s disease, elevated levels of ROS overwhelm the antioxidant defenses, leading to lipid peroxidation, protein oxidation, and DNA damage. Misfolded amyloid-beta proteins further exacerbate oxidative stress, creating a self-reinforcing cycle of damage.
2. Microglial activation and synaptic dysfunction: Chronic inflammation is a central feature of AD. Microglia activated by amyloid-beta deposits release pro-inflammatory cytokines, disrupting synaptic plasticity and accelerating neuronal loss.

### Viral influence on Alzheimer’s disease

Recent evidence suggests that viral infections may exacerbate oxidative stress, contributing to the onset or progression of Alzheimer’s disease. Viruses such as Herpes Simplex Virus type 1 (HSV-1) and Epstein–Barr virus (EBV) have been implicated in the pathological processes of Alzheimer’s disease (Itzhaki et al., 2016).

1 Mechanisms of Viral Contribution:

- *Viral reactivation*: HSV-1, when reactivated in the brain, can induce inflammation and oxidative stress, leading to neuronal dysfunction (Itzhaki et al., 2016).
- *Mitochondrial damage*: Viral proteins can directly impair mitochondrial function, exacerbating ROS production and energy deficits (Moon and Paek, 2015; Subramaniam and Chesselet, 2013).

2 *Amyloid-beta*: May act as an antimicrobial peptide, with chronic viral reactivation driving its pathological overproduction and aggregation (Guglielmotto et al., 2010).
3 *Herpes simplex virus and oxidative stress*: HSV-1 infection has been shown to trigger lipid peroxidation and protein oxidation, both of which contribute to membrane damage and neuronal death (Villa et al., 2020). The virus also promotes the aggregation of Aβ42, amplifying the oxidative stress cycle (Villa et al., 2020).
4 *Epstein–Barr virus and neuroinflammation*: EBV infection has been associated with chronic inflammation and microglial activation. Persistent infection can lead to elevated levels of pro-inflammatory cytokines, further disrupting synaptic plasticity. Beyond Alzheimer’s disease, EBV has also been strongly implicated in multiple sclerosis (MS), where chronic infection is now considered a major environmental trigger of neuroinflammation and demyelination (Bjornevik et al., 2022). Including MS in this context highlights the broader role of EBV as a driver of chronic neuroinflammatory processes that can contribute to neurodegenerative pathology. Beyond its potential role in Alzheimer’s disease, Epstein–Barr virus (EBV) has also been strongly implicated in multiple sclerosis (MS), where it is now considered a major environmental trigger of chronic neuroinflammation and demyelination.

Large longitudinal studies have shown that nearly all patients with MS have prior EBV infection, and seroconversion to EBV positivity increases the risk of developing MS more than 30-fold (Bjornevik et al., 2022). EBV is thought to establish latent infection in B cells, which can trigger autoimmune responses against myelin through molecular mimicry, abnormal antigen presentation, and sustained activation of pro-inflammatory cytokines. Chronic EBV activity in MS is associated with oligoclonal band production in cerebrospinal fluid, microglial activation, and infiltration of autoreactive T cells, all of which contribute to persistent neuroinflammation. Highlighting EBV’s role in MS strengthens the broader relevance of viral contributions to neurodegeneration, demonstrating how a chronic viral trigger can initiate and sustain a pathological cascade of inflammation and oxidative damage. This parallel suggests that antiviral therapies, already being explored in MS, could similarly complement preventive strategies for AD and PD in individuals with latent or reactivated viral infections. These findings reinforce the role of inflammation as both a driver and a modifiable factor in neurodegeneration. Our model integrates lithium’s anti-inflammatory effects with biomarker monitoring (e.g., YKL-40, sTREM2) to translate these insights into targeted preventive strategies.

**Figure:**
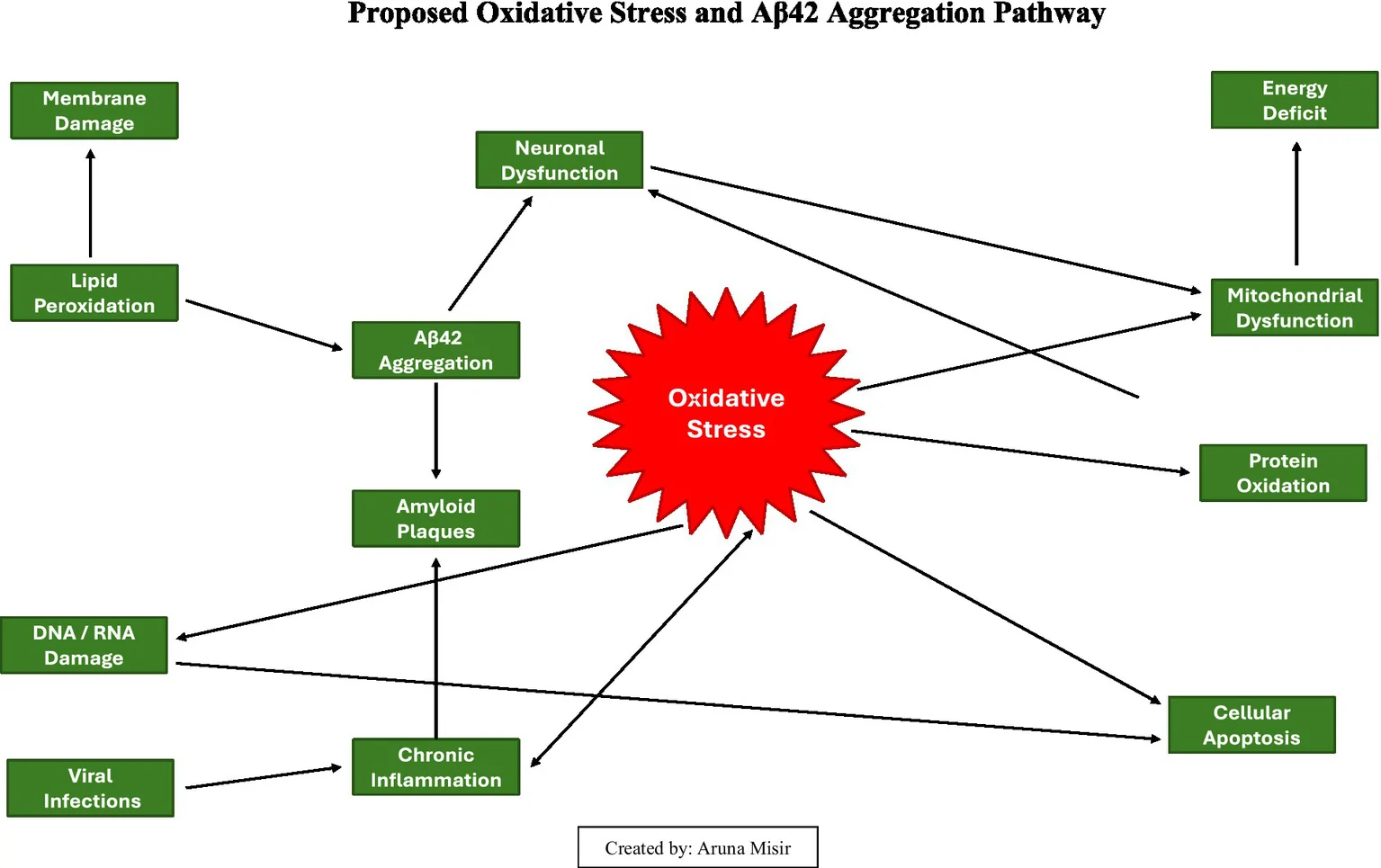


Diagram explanation: The accompanying diagram illustrates the pathophysiological mechanisms of Aβ42 aggregation, oxidative stress, and neurodegeneration in Alzheimer’s disease:

- Inducing oxidative stress through reactivation and inflammation (Itzhaki et al., 2016).
- Disrupting mitochondrial function and energy production (Dexter and Jenner, 2013).
- Enhancing amyloid-beta aggregation as part of the brain’s antimicrobial defense mechanism (Bjornevik et al., 2022).

*Appendix 1: Pathway Description*

1 *Oxidative stress*: Oxidative stress refers to the imbalance between reactive oxygen species (ROS) production and the body’s antioxidant defenses. It initiates a cascade of damaging events (Chapleau et al., 2022).
2 *Lipid peroxidation*: ROS attack lipids in cell membranes, leading to membrane damage and loss of neuronal integrity. (Hidisoglu et al., 2018).
3 *Protein oxidation*: ROS cause protein damage, disrupting neuronal signaling and synaptic function (Villa et al., 2020).
4 *DNA/RNA damage*: Oxidative stress damages genetic material, leading to cellular apoptosis (Mao and Reddy, 2011).
5 *Mitochondrial dysfunction*: Mitochondria, as major ROS producers, suffer damage, resulting in energy deficits and further ROS production (Dexter and Jenner, 2013).
6 *Aβ42 aggregation*: The production of Aβ42 peptides increases under oxidative stress, leading to their aggregation into amyloid plaques (Su et al., 2004).
7 *Amyloid plaques*: Aggregated Aβ42 peptides form plaques, which exacerbate oxidative stress and inflammation (Villa et al., 2020).
8 *Neuronal dysfunction*: Cumulative damage from lipid peroxidation, protein oxidation, and energy deficits impairs neuronal function (Hidisoglu et al., 2018).
9 *Cellular apoptosis*: Persistent damage results in programmed cell death, contributing to neurodegeneration (Finkel, 2011).
10 *Viral influence*: Viruses such as HSV-1 and EBV enhance these pathological pathways by reactivating inflammation, impairing mitochondria, and promoting amyloid aggregation (Itzhaki et al., 2016). These findings reinforce the role of inflammation as both a driver and a modifiable factor in neurodegeneration. Our model integrates lithium’s anti-inflammatory effects with biomarker monitoring (e.g., YKL-40, sTREM2) to translate these insights into targeted preventive strategies.

### The interconnection of homeostasis, inflammation, and oxidative stress

In summary, when homeostasis is maintained, inflammation and oxidative stress act as protective mechanisms to combat threats and repair damage. However, when disrupted, due to chronic stress, poor nutrition, or genetic predispositions, these mechanisms spiral out of control, leading to tissue damage and chronic disease (Chovatiya and Medzhitov, 2014).

**Figure:**
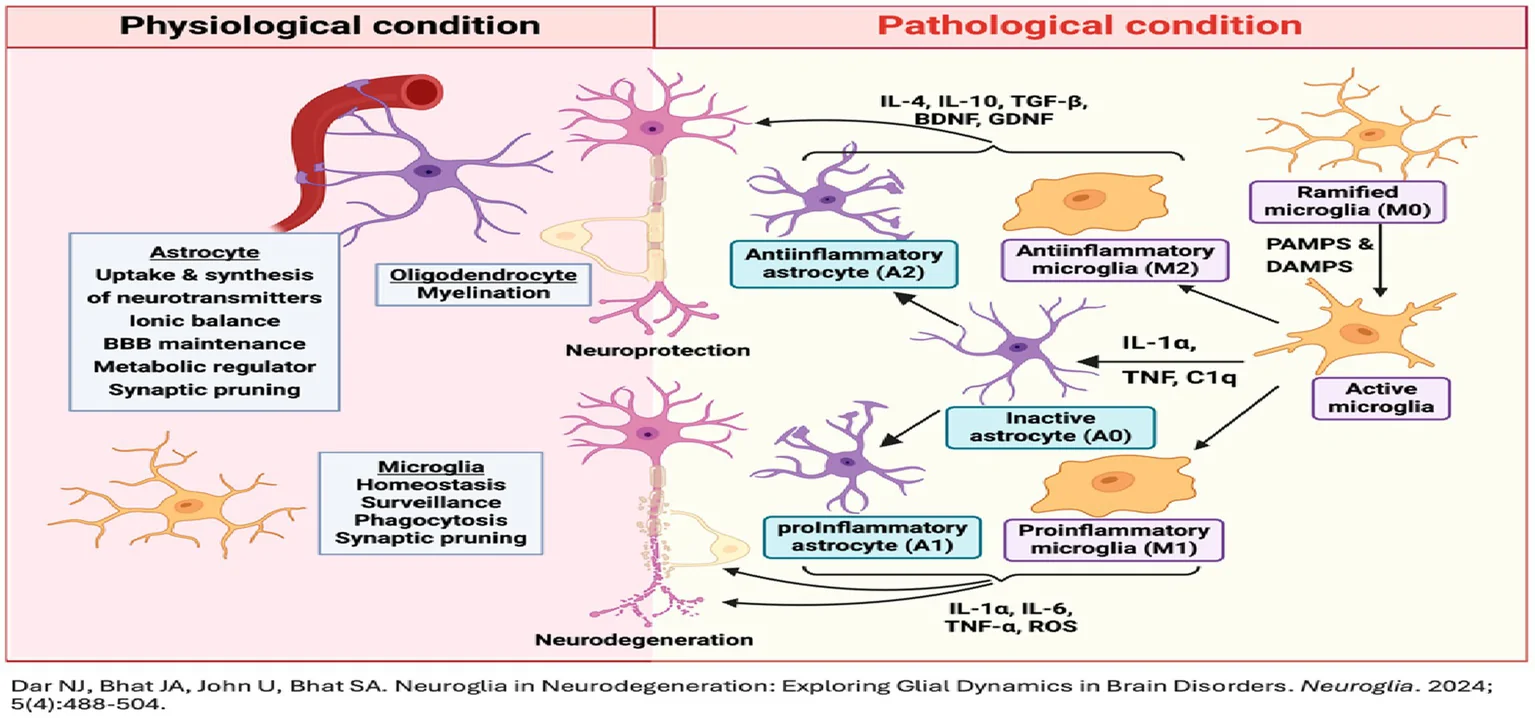


### Biochemical markers of body and brain

#### Glial fibrillary acidic protein – marker of astrocytic activation

- *Relevance*: GFAP is a cytoskeletal protein expressed in astrocytes, reflecting astrocytic activation in response to stress or injury. Elevated GFAP indicates blood–brain barrier (BBB) disruption, oxidative stress, and glial reactivity (Pelkmans et al., 2024).
- *In AD*: Increased GFAP is observed around amyloid plaques and correlates with astrocytic activation, oxidative burden, and disease severity (Pelkmans et al., 2024).
- *In PD*: GFAP levels rise in the substantia nigra, a region with high oxidative load. Chronic astrocytic activation in PD suggests insufficient compensation against oxidative stress and inflammation (Schulz et al., 2021).
- *Integration with lithium*: Lithium may attenuate astrocytic stress and lower GFAP levels by stabilizing inflammatory responses and promoting neurogenesis, although this has not yet been demonstrated directly. This suggests that GFAP may serve not only as a marker of disease progression, but also as a surrogate biomarker for monitoring lithium’s therapeutic response.

#### Neurofilament light chain – marker of neuronal injury

- *Relevance*: Neurofilament light chain (NfL) is a structural protein of axons that is released into cerebrospinal fluid (CSF) and blood when neurons undergo axonal injury. Elevated NfL levels serve as a sensitive and dynamic marker of neurodegeneration. Oxidative stress accelerates cytoskeletal breakdown and damages neuronal membranes, leading to the leakage of NfL into extracellular fluids. Thus, NfL reflects not only neuronal injury but also the ongoing progression of pathological processes in neurodegenerative disease. These same biomarkers are integral to our framework and suggest that PD, like AD, can be monitored using a common panel. Moreover, their modulation by lithium provides a translational pathway for preventive interventions across neurodegenerative disorders.
- *In AD*: NfL elevations correlate with tau pathology, synaptic loss, and cognitive decline (Jin et al., 2019). Longitudinal studies demonstrate that higher NfL levels predict faster cortical atrophy and conversion from mild cognitive impairment to Alzheimer’s disease (Villa et al., 2020; Jin et al., 2019). This makes NfL both a diagnostic biomarker and a tool for tracking disease trajectory.
- *In PD*: NfL levels rise as dopaminergic neurons in the substantia nigra degenerate, a process driven by oxidative damage and mitochondrial dysfunction. This positions NfL as a direct indicator of neuronal self-destruction and progression in PD.
- *Integration with lithium*: Lithium’s proposed role in reducing oxidative stress and stabilizing neuronal membranes suggests it could lower NfL levels by preventing further axonal damage and promoting repair mechanisms. Recent preclinical data provide strong support for this hypothesis. In a landmark *Nature* study (Aron et al., 2025), dietary lithium depletion in Alzheimer’s mouse models significantly worsened amyloid-*β* deposition, tau hyperphosphorylation, microglial activation, synaptic and myelin loss, and cognitive decline. Conversely, supplementation with lithium orotate prevented these pathological changes and preserved cognitive performance. This finding provides compelling evidence that lithium not only protects axonal integrity but may also directly modulate neuronal injury pathways reflected by NfL levels. Thus, NfL is a directly relevant biomarker for tracking lithium’s effect on axonal preservation and cognitive protection.

#### Soluble triggering receptor expressed on myeloid cells 2 – marker of microglial activation

- *Relevance*: sTREM2 is a biomarker of microglial activation, reflecting neuroinflammation. Chronic oxidative stress perpetuates microglial overactivation, leading to the release of pro-inflammatory cytokines and neuronal damage (Nordengen et al., 2019).
- *In AD*: Elevated sTREM2 accompanies amyloid plaque formation and tau aggregation, serving as a dynamic marker of microglial response and disease progression (Ma et al., 2020).
- *In PD*: sTREM2 levels are increased in the substantia nigra and correlate with microglial activation in regions of dopaminergic degeneration (Peng et al., 2019)
- *Integration with lithium*: Lithium exerts anti-inflammatory effects and may reduce microglial overactivation. Evidence suggests lithium modulates microglial signaling, potentially lowering sTREM2 and slowing neuroinflammation-driven neurodegeneration. Linking sTREM2 to lithium emphasizes how lithium may modulate microglial behavior and provides a marker for assessing its anti-inflammatory benefits.

#### YKL-40 (CHI3L1) – marker of neuroinflammation and tissue remodeling

- *Relevance*: YKL-40 is a glycoprotein secreted by activated astrocytes and microglia during inflammation and tissue remodeling. It reflects chronic neuroinflammation and extracellular matrix changes.
- *In AD*: Elevated YKL-40 levels correlate with amyloid plaque burden and neuroinflammation, tracking with cognitive decline and disease severity (Nordengen et al., 2019).
- *In PD*: YKL-40 increases in regions of glial activation and dopaminergic loss, reflecting the persistent inflammatory milieu in PD (Schulz et al., 2021).
- *Integration with lithium*: Lithium reduces neuroinflammation and promotes tissue repair. By stabilizing glial activity, lithium may lower YKL-40 levels and restore homeostasis, although this has not yet been tested directly. This makes YKL-40 a potential biomarker for evaluating lithium’s impact on glial function and repair processes.

#### S100B – marker of blood–brain barrier disruption and astrocytic activation

- *Relevance*: S100B is a calcium-binding protein secreted by astrocytes. Elevated levels indicate BBB disruption, oxidative stress, and astrocytic reactivity (Janigro et al., 2022).
- *In AD*: Increased S100B accompanies amyloid accumulation and gliosis, reflecting BBB compromise and glial overactivation (Merelli et al., 2021).
- *In PD*: Elevated S100B is found in areas of dopaminergic degeneration and correlates with astrocytic dysfunction and BBB leakage (Moon and Paek, 2015).
- *Integration with lithium*: Lithium may protect the BBB and modulate astrocytic activity. Evidence suggests lithium lowers S100B levels by attenuating astrocytic overactivation, thereby contributing to neuroprotection (Nassar and Azab, 2014). S100B therefore provides a readout of both pathology and lithium’s neuroprotective effect on the blood–brain barrier.

Taken together, these biomarkers: GFAP, NfL, sTREM2, YKL-40, and S100B, provide complementary insights into the astrocytic, axonal, microglial, and blood–brain barrier alterations that underlie neurodegenerative progression. Importantly, each is also influenced by lithium’s multifaceted actions, including inhibition of GSK-3β, stabilization of mitochondrial function, reduction of oxidative stress, and modulation of inflammatory signaling. This integration positions the biomarker panel not only as a diagnostic and prognostic tool, but also as a dynamic means of monitoring lithium’s preventive and therapeutic efficacy. By linking pathophysiological changes with measurable indicators of lithium’s effects, the model emphasizes a unified and translational approach to early detection and intervention in Alzheimer’s and Parkinson’s disease. Lithium’s inhibition of GSK-3β has been consistently demonstrated in both early and contemporary studies (Chen et al., 1999; Chiu et al., 2013), supporting its central role in modulating oxidative and inflammatory cascades.

Elevated levels of pro-inflammatory cytokines (e.g., IL-6, TNF-alpha) and oxidative stress markers (e.g., malondialdehyde) signal that homeostasis has been disrupted (Nathan and Ding, 2010). IL-6 is elevated across multiple conditions, including psychiatric disorders, autoimmune encephalitis, and neurodegeneration. While not specific to AD or PD, its elevation underscores a systemic pro-inflammatory state that may exacerbate existing oxidative and inflammatory processes.

**Figure:**
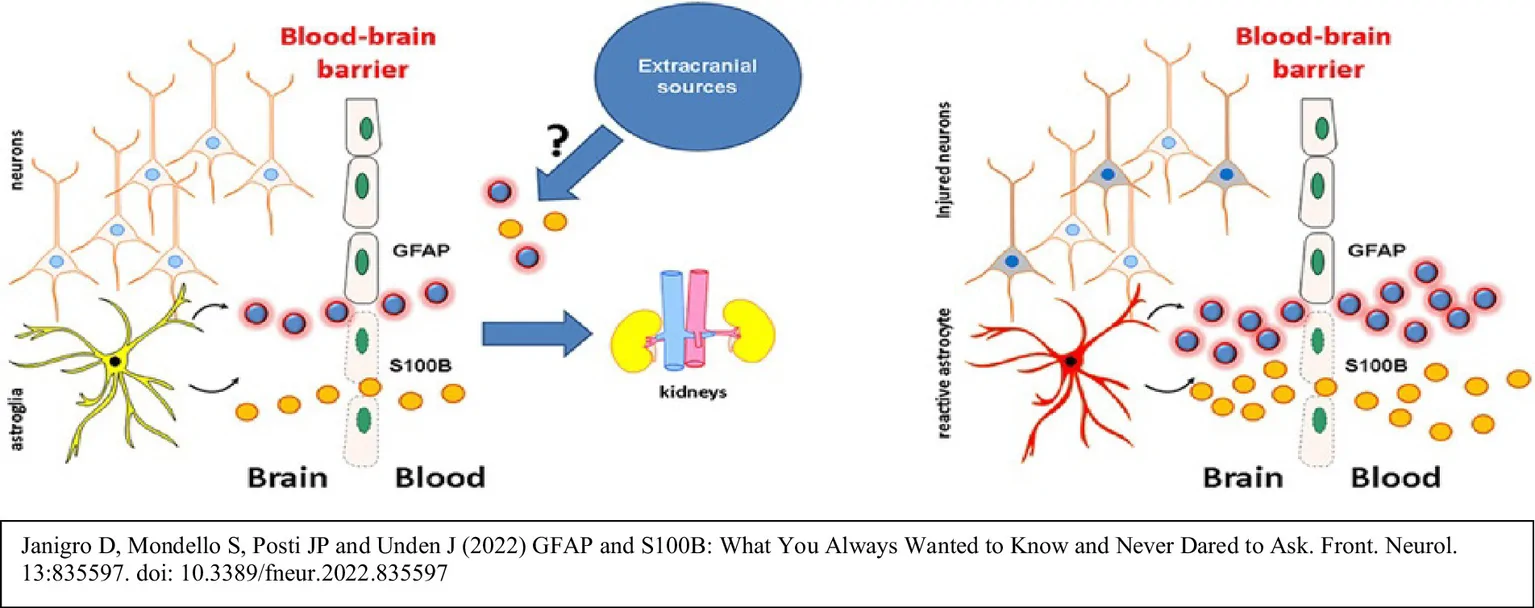


### Cellular sensors

Mitochondria detect metabolic stress and oxidative imbalance, triggering protective mechanisms like mitophagy (Pickles et al., 2018).

- The nuclear factor erythroid 2-related factor 2 (Nrf2) pathway activates during oxidative stress to upregulate detoxifying enzymes (Jiang et al., 2015).

### Neuroimmune communication

- The vagus nerve helps the brain modulate inflammatory responses, signaling anti-inflammatory activity when systemic inflammation is detected.

### Other early detection markers of oxidative stress

Early detection of oxidative stress is crucial for intervening before significant neuronal damage occurs in neurodegenerative diseases like Alzheimer’s and Parkinson’s. Several biomarkers have shown promise:

- *Malondialdehyde (MDA)*: A well-established marker of lipid peroxidation. Elevated MDA levels in plasma and cerebrospinal fluid (CSF) have been correlated with cognitive decline and disease severity in AD and PD (Butterfield and Halliwell, 2019; Mao and Reddy, 2011), supporting its potential role as a clinical indicator of early neurodegeneration.
- *4-Hydroxynonenal (4-HNE)*: A byproduct of lipid peroxidation that modifies proteins and nucleic acids. Increased levels of 4-HNE have been observed in the brains of AD patients and are associated with mitochondrial dysfunction and synaptic impairment (Guglielmotto et al., 2010; Mao and Reddy, 2011). Clinically, 4-HNE may provide insight into the oxidative environment that precedes overt neurodegenerative pathology.
- *Carbonylated proteins*: Protein carbonylation represents irreversible oxidative damage to cellular proteins. Elevated carbonylated proteins in blood and CSF are considered early predictors of neurodegeneration and correlate with both disease progression and severity (Luque-Contreras and Carvajal, 2014; Butterfield and Halliwell, 2019). This makes them useful as non-invasive biomarkers for tracking oxidative stress over time.
- *Mitochondrial activity markers*: Because mitochondria are the main source of ROS, impaired mitochondrial activity can be detected early and serves as a proxy for oxidative imbalance. Markers of mitochondrial dysfunction—including reduced activity of respiratory chain complexes and increased mitochondrial DNA damage—have been reported in both AD and PD (Schapira et al., 1990; Subramaniam and Chesselet, 2013; Moon and Paek, 2015).

Collectively, these biomarkers provide measurable indicators of oxidative stress that can be applied in both research and clinical settings. Their detection through high-precision assays in blood, CSF, or urine allows for identification of at-risk individuals before clinical symptoms appear.

### Detection methods

High-precision assays can be used to measure these biomarkers in cerebrospinal fluid (CSF), blood, or urine. Techniques such as ELISA, liquid chromatography–mass spectrometry (LC–MS), and immunoassays allow sensitive detection of malondialdehyde, 4-HNE, protein carbonyls, and mitochondrial activity markers. These methods have already been applied in clinical studies and can identify early oxidative stress imbalances, potentially predicting disease onset long before clinical symptoms appear (Villa et al., 2020; Butterfield and Halliwell, 2019; Mao and Reddy, 2011).

## Therapeutic approaches

1 Amyloid-beta modulation:

- Therapeutic strategies should focus on maintaining soluble Aβ42 levels to support synaptic plasticity while reducing toxic aggregates (Su et al., 2004).
- Monoclonal antibodies, such as Lecanemab, have shown potential in clearing amyloid plaques and lowering reactive oxygen species (ROS) production (Espay et al., 2022).

**Figure:**
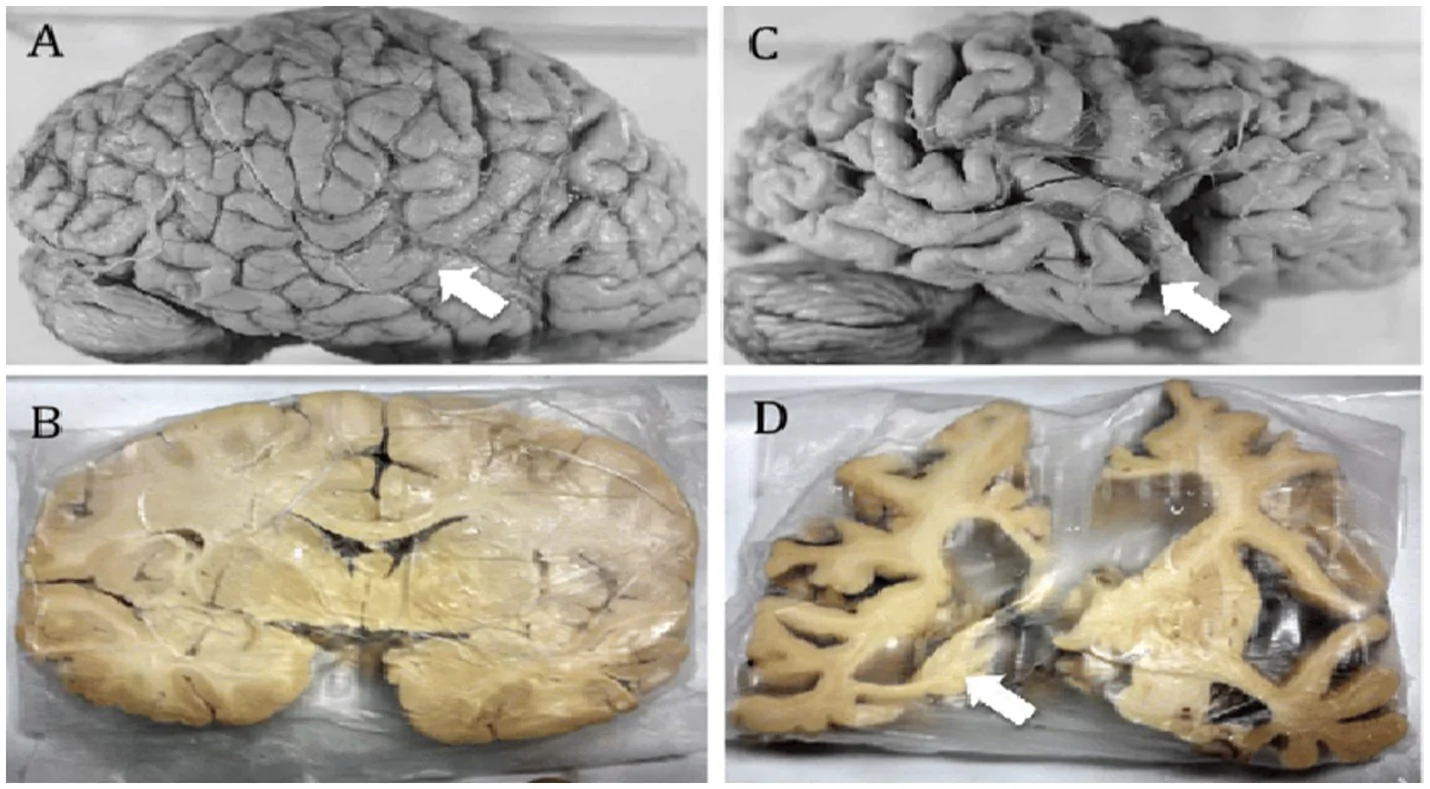

2 Antioxidant therapies:

In addition to amyloid-beta modulation, compounds with potent antioxidant activities can play a crucial role in slowing down neurodegenerative diseases by reducing oxidative stress and inflammation.

Examples include:

- *Rosmarinic acid*: A natural polyphenol with strong antioxidant properties, known to reduce lipid peroxidation and improve mitochondrial function (Hidisoglu et al., 2018).
- *Methylene blue*: Shown to enhance mitochondrial activity and reduce oxidative stress, methylene blue has demonstrated neuroprotective effects in preclinical models of Alzheimer’s and Parkinson’s disease (Tucker et al., 2018).
- *N-Acetylcysteine (NAC)*: A precursor to glutathione, NAC replenishes intracellular antioxidant defenses and mitigates oxidative damage (Bjelakovic et al., 2012).
- *Coenzyme Q10 (CoQ10)*: Essential for mitochondrial electron transport and energy production, CoQ10 supplementation has shown promise in improving mitochondrial function in neurodegenerative conditions (Dexter and Jenner, 2013).
- Curcumin: Known for its anti-inflammatory and antioxidant properties, curcumin can inhibit oxidative damage and amyloid plaque formation (Mao and Reddy, 2011).

### The role of Lithium in neurodegenerative: lithium’s role in combating oxidative stress

Oxidative stress is a central initiator of neurodegenerative processes. Lithium can directly modulate oxidative stress by:

- Enhancing mitochondrial health:

Mitochondria are major sources of reactive oxygen species (ROS). Dysfunctional mitochondria contribute to oxidative stress, neuronal energy failure, and cell death. Lithium stabilizes mitochondrial membranes, improves ATP production, and lowers ROS generation, directly addressing the oxidative stress query (Quiroz et al., 2010; Manji et al., 1999).

- Upregulating antioxidant defenses:

Lithium stimulates the production of key antioxidant enzymes such as glutathione peroxidase and superoxide dismutase (SOD). These enzymes neutralize ROS, preventing oxidative damage to neuronal membranes and DNA (Hashimoto et al., 2002).

### Lithium as a regulator of inflammation

Lithium’s anti-inflammatory effects are well-documented:

- *Inhibition of pro-inflammatory cytokines*: Lithium reduces levels of pro-inflammatory cytokines such as IL-1β, TNF-*α*, and IL-6, which are elevated in neuropsychiatric disorders. By downregulating these cytokines, lithium helps break the feedback loop of oxidative stress and inflammation (Nathan and Ding, 2010). IL-6 is elevated across multiple conditions, including psychiatric disorders, autoimmune encephalitis, and neurodegeneration. While not specific to AD or PD, its elevation underscores a systemic pro-inflammatory state that may exacerbate existing oxidative and inflammatory processes.
- *Modulation of microglial activation*: Chronic activation of microglia (the brain’s immune cells) leads to a state of persistent inflammation and further neuronal damage. Lithium prevents overactivation of microglia, thereby reducing the inflammatory cascade that drives neurodegeneration (Wang et al., 2024). Recent analyses by Hamstra et al. (2023) describe how low-dose lithium may attenuate neuroinflammation by modulating microglial activation states and promoting tissue repair, offering mechanistic support for its integration into preventive frameworks.

### Lithium and neuroplasticity: prevention of brain circuit destruction

A cornerstone of my theory is that oxidative stress disrupts neuroplasticity, leading to faulty brain circuitry and impaired synaptic communication. Lithium’s role in promoting neuroplasticity and neurogenesis:

- *Stimulation of neurogenesis*: Lithium increases the proliferation of neural progenitor cells in regions such as the hippocampus, which is critical for maintaining cognitive function. This could counteract the neurodegenerative effects seen in schizophrenia, where hippocampal atrophy is common (Malhi et al., 2013).
- *Upregulation of brain-derived neurotrophic factor (BDNF)*: BDNF plays a key role in supporting synaptic plasticity and neuronal survival. *Lithium enhances BDNF expression*, ensuring that neurons remain functional and capable of adapting to stressors (Hashimoto et al., 2002).
- *Prevention of synaptic loss*: In schizophrenia, loss of synaptic connections is a hallmark feature. Lithium has been shown *to preserve synaptic integrity by modulating NMDA receptor activity* and preventing excitotoxicity, a process driven by oxidative stress that leads to synaptic pruning (Nassar and Azab, 2014).

### Lithium and GSK-3*β* inhibition - a master regulator in my model

**Figure 1:**
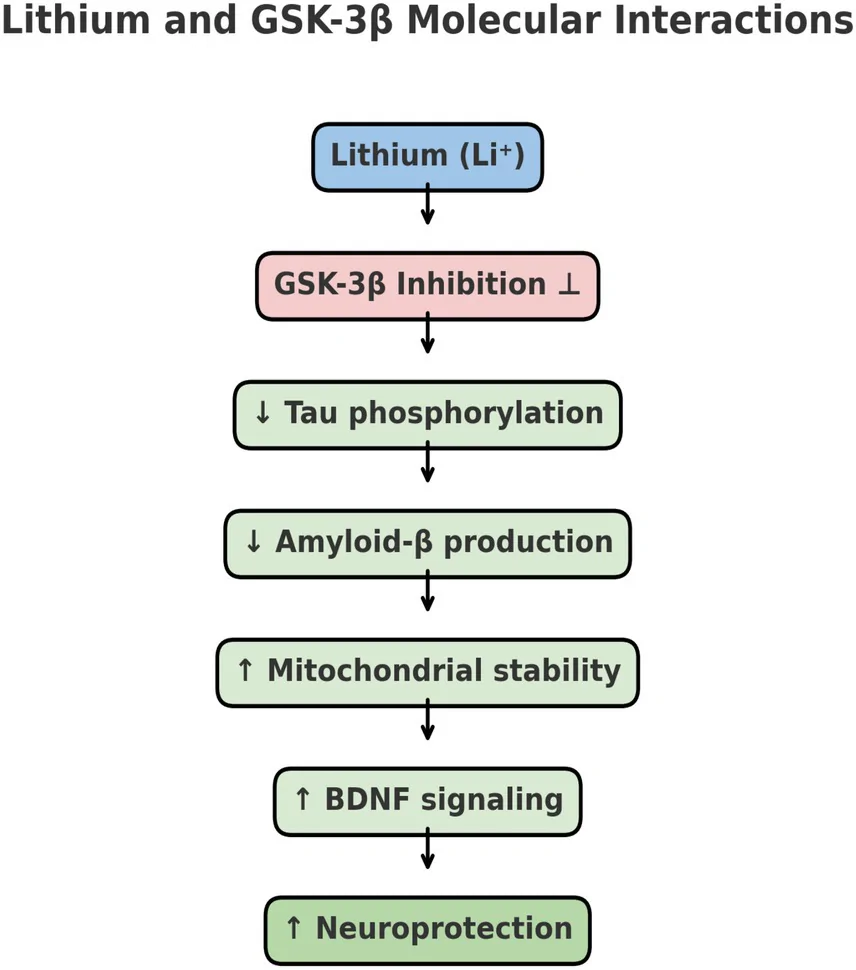
Schematic illustration of lithium’s molecular interactions with GSK-3β and downstream pathways. Lithium inhibits glycogen synthase kinase-3β (GSK-3β), a key enzyme implicated in tau phosphorylation (Engel et al., 2006; Forlenza et al., 2014). And amyloid-β production (Phiel et al., 2003; Su et al., 2004). Through this inhibition, lithium reduces tau hyperphosphorylation and amyloid accumulation, while simultaneously enhancing mitochondrial stability and reducing oxidative stress and increasing brain-derived neurotrophic factor (BDNF) signaling. Together, these effects converge to promote neuroprotection, supporting lithium’s potential role as a disease-modifying and preventive therapy in neurodegenerative disorders. While each of these mechanistic pathways has been described individually in the literature, to our knowledge they have not previously been presented in a single integrated scheme. This figure therefore emphasizes the convergence of lithium’s multifaceted actions into one unified model, highlighting its relevance to preventive strategies in Alzheimer’s disease and Parkinson’s disease.

Lithium’s pleiotropic actions extend beyond GSK-3β inhibition; early work by Chen et al. (1999) demonstrated its direct suppression of GSK-3β activity in intact cells, while Chiu et al. (2013) and Forlenza et al. (2014) showed that lithium exerts broad neuroprotective, anti-inflammatory, and mitochondrial-stabilizing effects. My theory highlights how molecular dysregulation due to oxidative stress can activate pathological signaling pathways. Lithium’s inhibition of Glycogen Synthase Kinase-3 Beta (GSK-3β) is particularly relevant:

- *Role of GSK-3β in neurodegeneration*: Overactivation of GSK-3β leads to increased oxidative stress, tau hyperphosphorylation (associated with Alzheimer’s), and neuronal apoptosis. In schizophrenia, GSK-3β dysregulation has been linked to impaired neuroplasticity and altered neurotransmitter signaling (Jope and Johnson, 2004).
- *Lithium as a GSK-3β inhibitor*: By inhibiting GSK-3β, Lithium prevents these destructive processes, helping to maintain neuronal health and stability. This directly supports my hypothesis that lithium deficiency removes a key regulatory mechanism, allowing oxidative stress and inflammation to spiral out of control (Phiel and Klein, 2001). This positions lithium uniquely compared to current therapeutic options, since its multi-targeted effects link directly to the biomarkers we propose, allowing prevention to be tracked and validated in clinical settings (Figure 1).

### Lithium in Parkinson’s and Alzheimer’s diseases

Early epidemiological work suggested geographic differences in lithium exposure may influence mental health outcomes, laying groundwork for later biological studies (Dawson et al., 1970). While lithium has historically been studied as a mood stabilizer in psychiatric disorders, accumulating evidence highlights its broader role as a neuroprotective agent across neurodegenerative diseases. Importantly, a recent *Nature* study from Harvard (2025) demonstrated that lithium is an essential micronutrient for brain health. In Alzheimer’s mouse models, dietary lithium depletion exacerbated amyloid-*β* and tau pathology, microglial activation, and cognitive decline, whereas supplementation with lithium orotate prevented these changes and preserved neuronal function. These finding bridges psychiatric and neurodegenerative context, supporting a unified framework in which lithium deficiency contributes to neuronal vulnerability, while restoration of physiological lithium levels provides resilience. Ecological studies provide additional context, showing that regions with naturally higher lithium concentrations in drinking water exhibit lower rates of psychiatric hospitalization and violent behavior (Dawson et al., 1970; Schrauzer and Shrestha, 1990), supporting the concept that trace lithium exposure may play a stabilizing neurobiological role.

- *Parkinson’s disease (PD)*: In PD, progressive loss of dopaminergic neurons in the substantia nigra leads to motor symptoms such as tremors and rigidity. Oxidative stress and mitochondrial dysfunction are central to PD pathology. Lithium’s ability to enhance mitochondrial function, reduce ROS, and inhibit GSK-3β has shown promise in preclinical models of PD, where it helps protect dopaminergic neurons from oxidative damage (Nassar and Azab, 2014). Additionally, lithium has been found to modulate autophagy, a cellular process critical for clearing damaged organelles and proteins, which is impaired in PD (Wang et al., 2024).
- *Alzheimer’s disease (AD)*: AD is characterized by the accumulation of beta-amyloid plaques and neurofibrillary tangles composed of hyperphosphorylated tau protein. Lithium’s inhibition of GSK-3β reduces tau hyperphosphorylation, thereby decreasing the formation of neurofibrillary tangles (Jope and Johnson, 2004). Moreover, lithium has been shown to decrease beta-amyloid production by modulating secretase activity, reducing plaque formation (Phiel et al., 2003). Clinical studies have also suggested that long-term lithium use may reduce the risk of developing dementia, further supporting its potential role in AD prevention (Malhi et al., 2013).

Lastly, converging preclinical and clinical evidence highlights lithium’s potential to preserve cognition in Alzheimer’s disease. In a clinical trial, patients receiving lithium showed stable cognitive and functional scores after 24 months, whereas the placebo group demonstrated slightly but significantly worsened scores. Although no significant differences were observed in hyperphosphorylated tau or total tau protein concentrations, the lithium group exhibited a trend toward diminished conversion into Alzheimer’s disease (Rybakowski, 2022).

**Figure:**
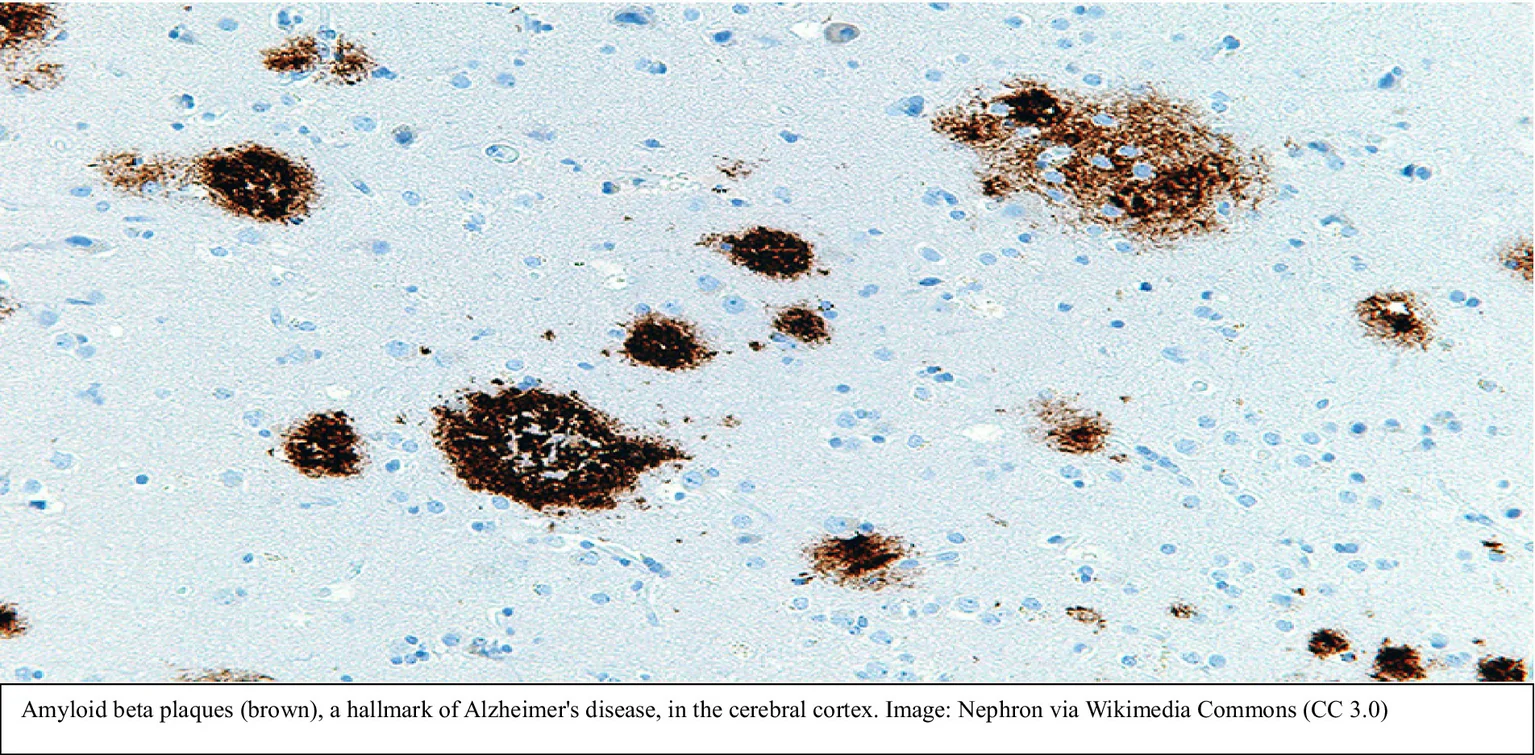


Complementing these clinical findings, a groundbreaking *Nature* study by Harvard researchers (2025) demonstrated that reducing dietary lithium by more than 50% in Alzheimer’s mouse models accelerated amyloid-*β* and tau pathology, increased microglial activation, and led to synaptic loss and cognitive decline. Remarkably, lithium orotate supplementation prevented these changes and preserved neuronal and cognitive function (Aron et al., 2025). Together, these results establish a compelling continuum of evidence from animal models to clinical trials, supporting lithium as both a neuroprotective agent and a potential preventive therapy in neurodegenerative disease. Lithium’s neuroprotective effects have been demonstrated across multiple preclinical and clinical studies, showing reductions in oxidative stress and tau phosphorylation (Forlenza et al., 2014).

### Safety considerations and therapeutic window of Lithium

While the neuroprotective potential of lithium is increasingly supported by both clinical and preclinical studies, it is essential to recognize its dose-dependent risks. Long-term use of pharmacological doses has been associated with adverse effects including renal impairment, hypothyroidism, and tremor (Malhi et al., 2013); However, emerging evidence, including the recent Harvard *Nature* study (2025) suggests that lithium at physiological or trace levels is necessary for maintaining neuronal integrity, and that deficiency itself may be pathogenic, accelerating amyloid and tau pathology as well as cognitive decline. This perspective reframes lithium not solely as a drug, but as a vital micronutrient with a therapeutic window, where low-dose supplementation may confer preventive benefits without the toxicities seen at higher clinical doses. Such an approach supports the concept of lithium as both a nutrient and a neuroprotective agent, aligning with our preventive treatment model.

**Figure:**
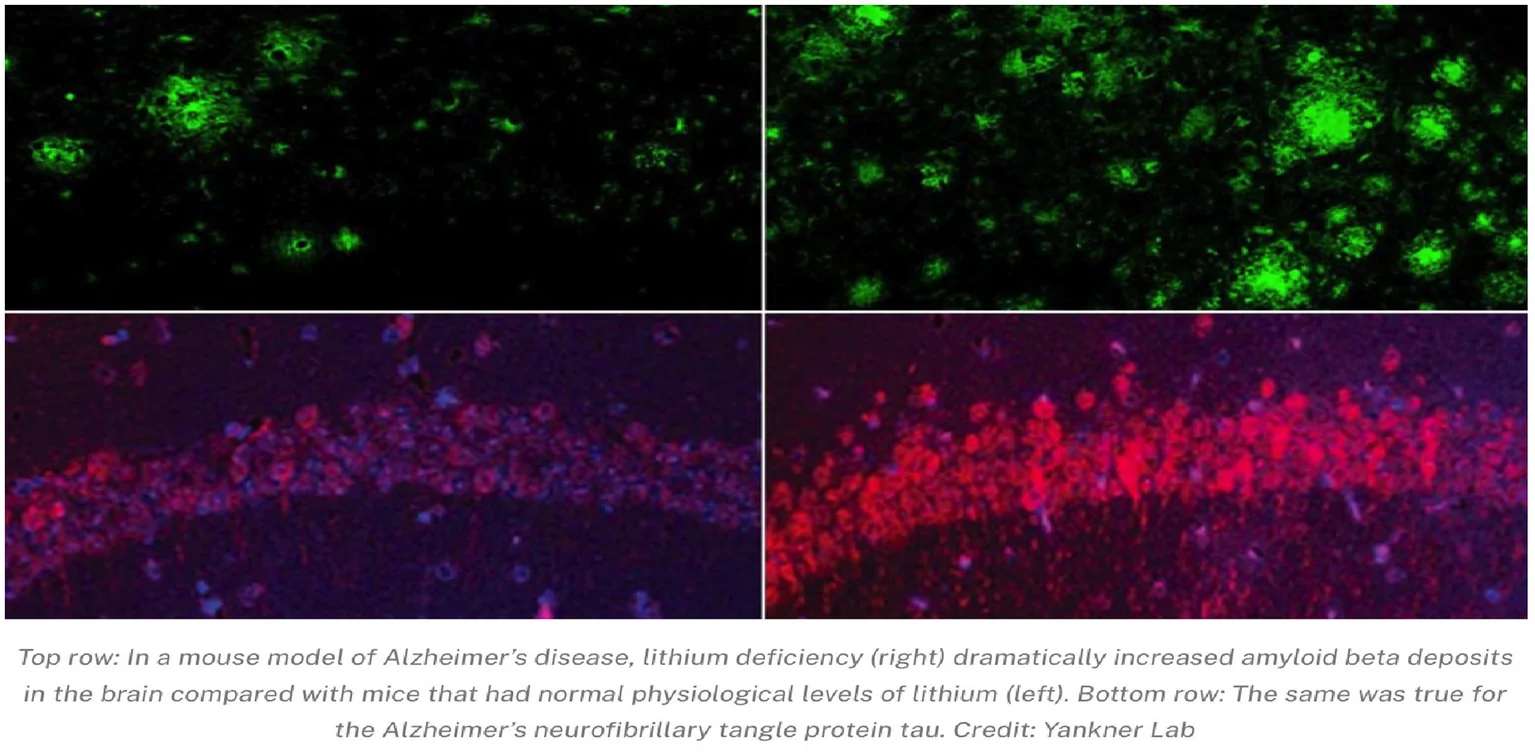


**Figure:**
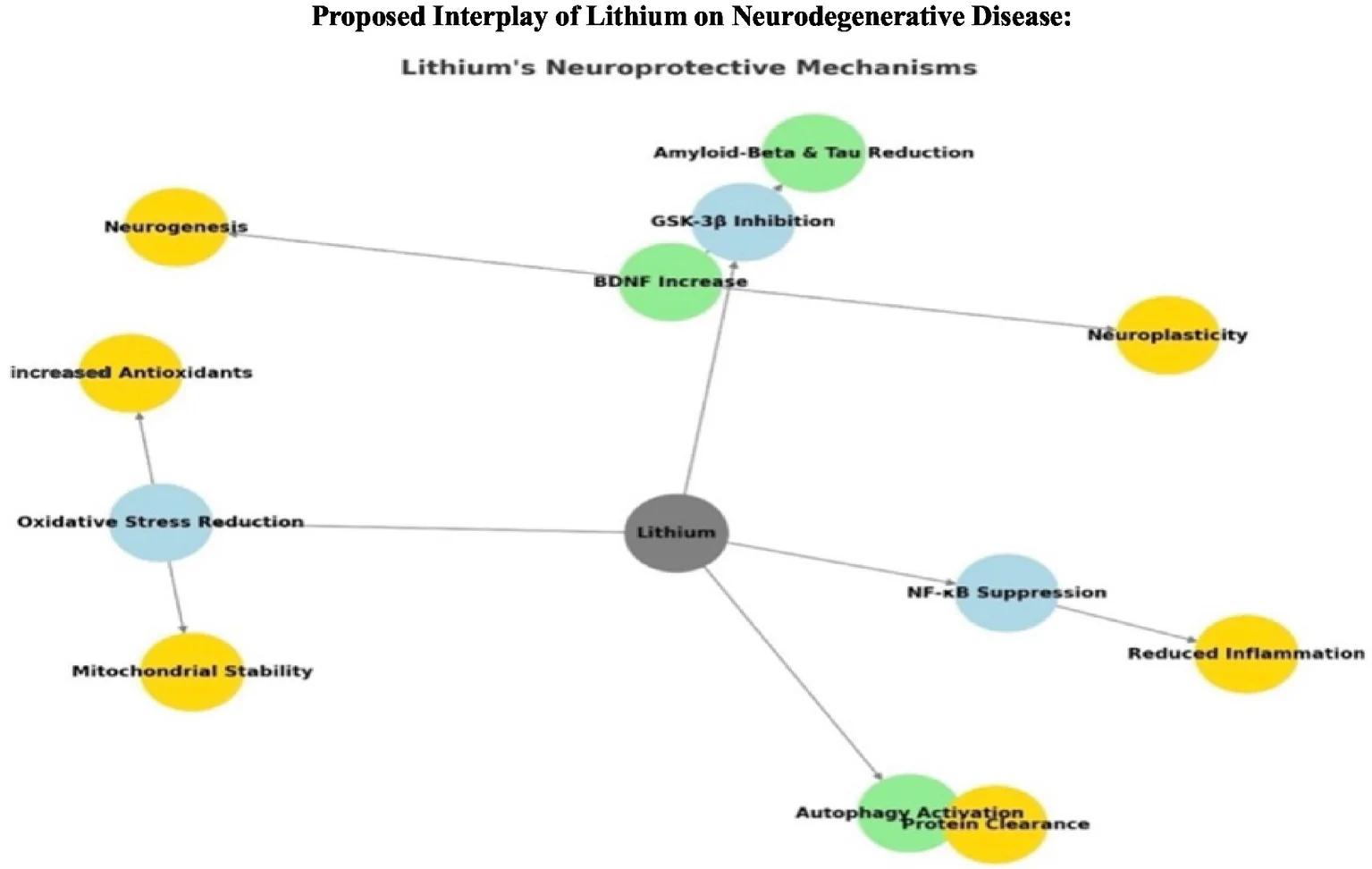


### Lithium as an essential micronutrient: insights from the Harvard nature study

By demonstrating that lithium deficiency itself is what triggers amyloid and tau pathology, the Harvard Nature 2025 study offers powerful justification for the central premise of my theory. Recent evidence from a landmark study conducted by Harvard Medical School researchers, published in *Nature* (2025), provides compelling support for lithium’s fundamental role in brain health. In this work, Yankner and colleagues demonstrated that lithium is not merely a pharmacological agent but a naturally occurring endogenous micronutrient in the brain that is essential for preserving neuronal function and preventing neurodegeneration.

Using both wild-type mice and Alzheimer’s disease (AD) mouse models, the study showed that reducing dietary lithium levels by approximately 50% accelerated the development of AD-like pathology including:

- Increased amyloid-β deposition and tau hyper-phosphorylation
- Microglial activation and heightened neuroinflammation
- Synaptic and myelin loss
- Progressive cognitive decline

Mechanistically, these effects were linked to dysregulated glycogen synthase kinase-3β (GSK-3β) activity, a pathway strongly implicated in both AD and other neurodegenerative disorders. Strikingly, supplementation with lithium orotate, a salt formulation capable of bypassing amyloid plaque binding, prevented these pathological changes. Mice receiving lithium orotate exhibited preserved synaptic integrity, reduced microglial activation, and maintained cognitive performance. Recent reviews emphasize that low-dose lithium supplementation may support neuronal resilience even outside psychiatric settings, reinforcing its broader biological role (Hamstra et al., 2023).

This study provides critical validation of our model in several ways:

1. It demonstrates that lithium deficiency is not neutral but pathogenic, amplifying oxidative stress, inflammation, and neurodegenerative cascades.
2. It supports our argument that restoring physiological lithium levels may prevent the biomarker changes we describe, including elevations of NfL and GFAP.
3. It reinforces lithium’s dual role as both a nutrient and a therapeutic agent, uniting nutritional neuroscience with clinical neuroprotection.

Together with existing evidence on lithium’s ability to stabilize mitochondrial function, reduce oxidative stress, and promote neuroplasticity, the Harvard findings firmly situate lithium as a cornerstone in the prevention and treatment of neurodegenerative disease.

### Implications for treatment

My theory suggests that lithium supplementation, particularly in trace amounts, could serve as a preventive strategy neurodegenerative disease by:

- *Restoring oxidative balance*: Providing exogenous lithium could help restore the brain’s natural antioxidant defenses, preventing the cascade of oxidative damage (Quiroz et al., 2010).
- *Enhancing circuit repair*: Long-term low-dose lithium therapy could enhance neuroplasticity, enabling the brain to repair dysfunctional circuits and prevent disease progression (Malhi et al., 2013).
- *Preventing early neurodevelopmental damage*: If oxidative stress begins early in life, lithium supplementation during critical developmental windows may prevent the initial damage that predisposes individuals to neurodegenerative diseases.

These antioxidants and lithium either alone or in combination with other therapies like TMS, represent promising avenues for neuroprotection and disease modification. Further clinical research is needed to validate their efficacy in large-scale trials (Malhi et al., 2013).

### Role of transcranial magnetic stimulation in Alzheimer’s disease

TMS has been explored as a non-invasive modality to slow cognitive decline in Alzheimer’s disease. A landmark study demonstrated that rTMS combined with cognitive training significantly improved cognitive performance in Alzheimer’s disease patients. These findings suggest that TMS may enhance neuroplasticity and strengthen synaptic connections, counteracting some of the disease’s effects. Systematic reviews further confirm that rTMS can improve global cognitive function, particularly when used as part of a multimodal treatment strategy. The role of transcranial magnetic stimulation (TMS) in modulating oxidative stress and enhancing cognitive function has been increasingly recognized. In Alzheimer’s disease, repetitive TMS has been shown to reduce oxidative stress biomarkers, improve neuroplasticity, and strengthen connectivity between parietal and hippocampal regions (Bashir et al., 2022; Velioglu et al., 2021). These findings support the inclusion of TMS in my model as a therapeutic modality that directly complements antioxidant strategies.

In their 2022 review, Bashir et al. discuss the potential of transcranial magnetic stimulation (TMS) to induce neurobiological changes in Alzheimer’s disease (AD), particularly focusing on its effects on oxidative stress. They reference a study by Velioglu et al., which applied 20 Hz. repetitive TMS (rTMS) to the lateral parietal cortex of AD patients. This intervention led to increased levels of brain-derived neurotrophic factor (BDNF), total antioxidant status, total thiol, and native thiol. Concurrently, there were decreases in total oxidant status, oxidative stress index, oxidant enzyme activity, and disulfide levels.

These findings suggest that rTMS may reduce oxidative stress in the AD brain, potentially contributing to neuroprotection and cognitive improvement in their 2021 study, Velioglu et al. explored the therapeutic effects of 20 Hz repetitive transcranial magnetic stimulation (rTMS) applied to the left lateral parietal cortex in patients with Alzheimer’s disease (AD). The study provided compelling evidence that rTMS produces both cognitive and biological improvements, particularly through reducing oxidative stress and enhancing neurotrophic support. Early clinical work showed that repetitive TMS could improve cognitive performance in Alzheimer’s disease, laying the groundwork for later mechanistic studies (Rabey et al., 2013).

### Key findings

1 *Cognitive function improvement*: Patients showed significant improvement in cognitive performance, particularly in visual recognition memory and the clock-drawing test. These tasks are sensitive to early cognitive decline in AD, and the observed improvement points to enhanced parietal-hippocampal interaction: a relationship critical to memory and visuospatial function.
2 *Increase in BDNF levels*: BDNF (brain-derived neurotrophic factor) is vital for neuronal survival, synaptic plasticity, and neurogenesis. The rTMS treatment led to a measurable increase in BDNF, suggesting a neuroprotective and regenerative mechanism was activated.
3 *Reduction in oxidative stress*: One of the most profound findings was the reduction of oxidative stress biomarkers, which are typically elevated in AD and contribute to neuronal degeneration. Post-rTMS treatment results showed:

- Increased total antioxidant status (TAS)
- Increased total and native thiol levels
- Decreased total oxidant status (TOS)
- Decreased oxidative stress index (OSI)
- Reduced disulfide levels and oxidant enzyme activity

4 *Enhanced resting-state brain connectivity*: Functional MRI results revealed enhanced connectivity between the stimulated lateral parietal cortex and the hippocampus, which is crucial for episodic memory. This neural network remodeling supports the idea that TMS is not just symptomatic; it may encourage reparative changes in brain circuitry. This shift toward a more antioxidative biochemical profile suggests rTMS actively counteracts one of the major neurodegenerative pathways in Alzheimer’s disease.

*Parkinson’s disease*: The silent erosion of motor function: Parkinson’s disease is a progressive disorder characterized by the loss of dopaminergic neurons in the substantia nigra, a region of the brain critical for motor control. Importantly, Parkinson’s disease does not begin with tremor and stiffness alone. Many patients first present with prodromal symptoms years before motor onset, including anosmia (loss of smell), REM sleep behavior disorder, and autonomic dysfunction such as constipation and orthostatic hypotension. These early features are increasingly recognized as clinical harbingers of PD and are closely aligned with underlying oxidative and inflammatory changes. The presence of such non-motor symptoms supports the concept that PD is a systemic process, with neurodegeneration beginning long before classical motor signs become apparent (Hirsch and Hunot, 2009; Merelli et al., 2021).

**Figure:**
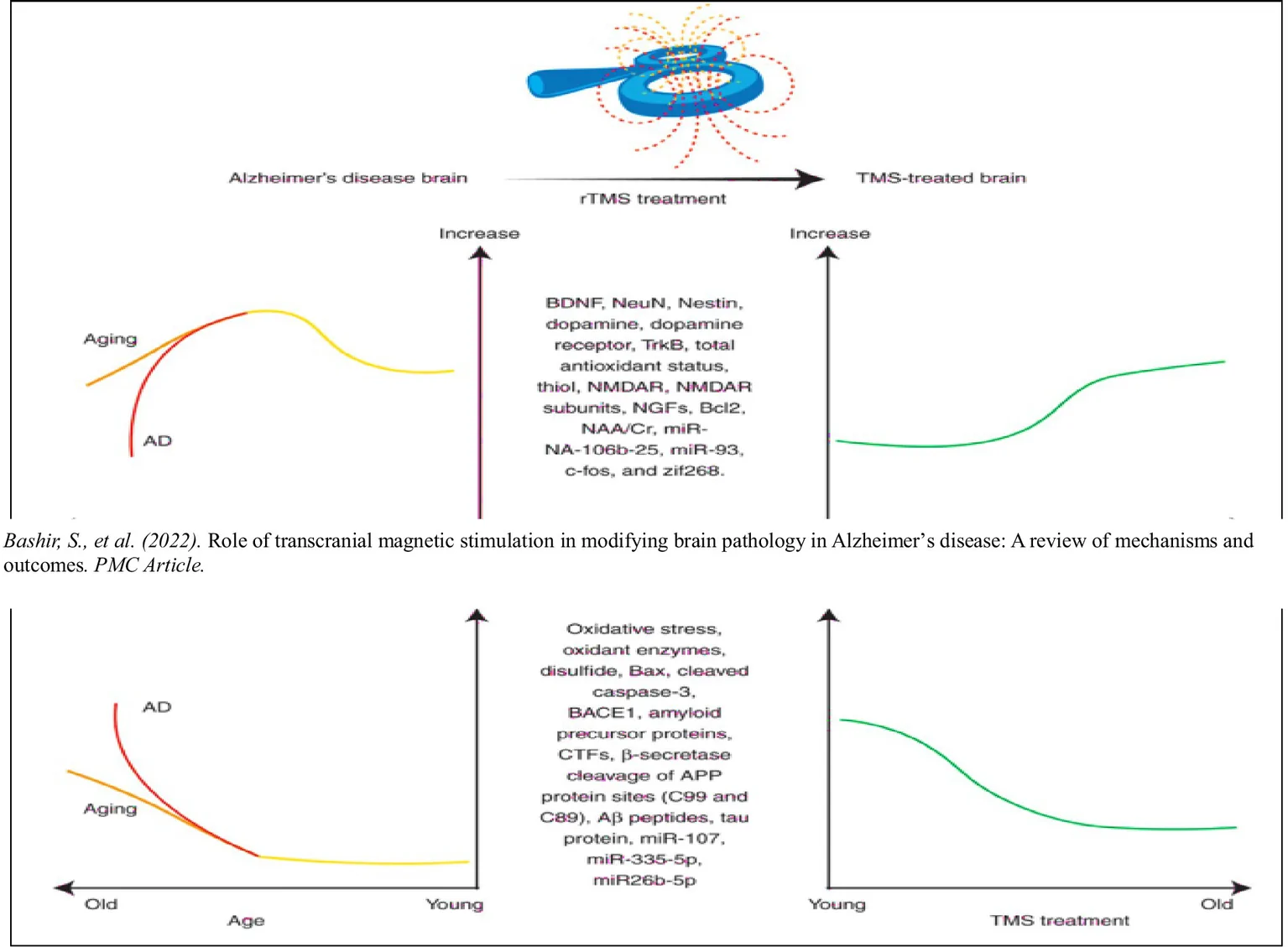

1. *Oxidative stress and inflammation in Parkinson’s disease*: Dopamine metabolism itself contributes to oxidative stress, producing reactive oxygen species (ROS) that damage surrounding cells. The substantia nigra is particularly vulnerable due to its high metabolic demand and relatively low antioxidant defenses (Bjornevik et al., 2022). Compounding this is mitochondrial dysfunction, impairment in the cellular powerhouses that produce energy, leading to further ROS generation and neuronal injury (Dexter and Jenner, 2013). This finding is directly relevant to our framework, since lithium’s capacity to reduce ROS generation and stabilize mitochondrial function may counteract precisely this oxidative vulnerability in dopaminergic neurons.
2. *Microglial activation*: In PD, microglia, the brain’s immune cells, become chronically activated, releasing pro-inflammatory cytokines like tumor necrosis factor-alpha (TNF-*α*) and interleukin-1β (IL-1β). The chronic inflammation then exacerbates oxidative stress, creating a cycle of neuronal damage that underpins the disease’s progression (Tansey et al., 2007).
3. *Role of transcranial magnetic stimulation in Parkinson’s disease*: Repetitive transcranial magnetic stimulation (rTMS) has emerged as a promising tool for modulating neural activity in PD. High-frequency rTMS applied to the motor cortex has shown significant improvements in motor symptoms such as bradykinesia and rigidity. Additionally, studies suggest that rTMS may alleviate non-motor symptoms, including depression and cognitive dysfunction, which are common in PD (Machado-Vieira et al., 2009). Recent protocols combining rTMS with personalized functional reserve assessments have demonstrated potential in enhancing ambulatory function, highlighting the importance of tailoring interventions to individual patient needs (Velioglu et al., 2021). This aligns with our multimodal approach, in which TMS works synergistically with lithium and antioxidants to reduce oxidative stress, enhance neuroplasticity, and provide functional improvements in both motor and cognitive domains.

### Combining TMS and antioxidant therapies: a vision for the future

The integration of TMS with antioxidant therapies represents a new frontier in the treatment of neurodegenerative diseases. Oxidative stress plays a pivotal role in the pathogenesis of both PD and AD, and addressing this through targeted interventions could amplify the benefits of TMS (Bashir et al., 2022).

### Synergistic potential of TMS and antioxidants

Emerging evidence suggests that transcranial magnetic stimulation (TMS) and antioxidants may act synergistically in mitigating neurodegeneration. Antioxidants reduce oxidative stress and lipid peroxidation, thereby stabilizing the cellular environment and improving neuronal responsiveness to external stimulation. By lowering oxidative burden, antioxidants may enhance the efficacy of TMS in inducing synaptic plasticity and restoring network connectivity. Conversely, TMS has been shown to upregulate neurotrophic factors, such as BDNF, and improve functional connectivity between cortical and hippocampal regions, changes that can amplify the protective effects of antioxidant compounds (Bashir et al., 2022). Clinical studies also support this interaction: in Parkinson’s disease, rTMS improved cognition, mood, and walking ability (Wang et al., 2024), benefits that could be potentiated in the presence of optimized redox balance. Together, these findings indicate that the combined use of TMS and antioxidants may offer superior neuroprotection compared with either modality alone, underscoring the promise of multimodal therapy in Alzheimer’s and Parkinson’s disease.

1 Potential synergistic effects

- *Neuroprotection*: Antioxidants such as coenzyme Q10 and N-acetylcysteine may protect neurons from oxidative damage, while TMS enhances neural plasticity and functional connectivity (Dexter and Jenner, 2013).
- *Symptom management*: TMS can alleviate motor and cognitive symptoms, while antioxidants address the underlying oxidative stress, providing more comprehensive relief (Malhi et al., 2013).

2 *Disease modification*: The combined approach could slow or alter disease progression more effectively than either modality alone (Wang et al., 2024).
3 *Clinical trials and emerging evidence*: A clinical trial investigating the effects of rTMS combined with treadmill training in PD patients has shown promising results, paving the way for further research into multimodal approaches (Velioglu et al., 2021). While current studies focus primarily on motor and cognitive symptoms, future trials integrating antioxidants could yield groundbreaking insights into disease modification.

### Limitations and challenges

While the proposed framework integrates biomarkers, oxidative stress mechanisms, and multimodal therapies into a preventive strategy, several limitations must be acknowledged.

1. *Variability of antioxidant trials*: Clinical studies of antioxidant compounds have shown inconsistent outcomes, with some demonstrating neuroprotective effects and others reporting little benefit (Bjelakovic et al., 2012). This variability may reflect differences in dosage, bioavailability, patient selection, or trial design. Further standardized studies are needed to establish efficacy.
2. *Accessibility and cost of TMS*: Repetitive transcranial magnetic stimulation (rTMS) is a promising non-invasive intervention; however, its clinical use remains limited by cost, specialized equipment, and the need for trained personnel (Bashir et al., 2022; Wang et al., 2024). These barriers may reduce feasibility for large-scale preventive applications.
3. *Lithium’s therapeutic window*: Although trace or low-dose lithium supplementation appears safe and potentially protective, long-term pharmacological doses are associated with adverse effects, including renal impairment, thyroid dysfunction, and tremor (Malhi et al., 2013); The recent *Nature* study (Aron et al., 2025), which demonstrated that lithium deficiency exacerbates amyloid and tau pathology while supplementation prevented decline in mouse models of AD, provides strong support for the biological plausibility of our model. However, translating these findings to humans will require carefully designed trials to balance efficacy with safety.
4. *Biomarker specificity and sensitivity*: While biomarkers such as GFAP, NfL, sTREM2, and YKL-40 show promise for early detection, each lacks absolute disease specificity. Many are elevated in multiple neurological and systemic conditions, and assay variability remains a challenge (Forlenza et al., 2014). Longitudinal validation in large cohorts is needed to establish predictive value.
5. *Implementation in clinical practice*: Even if validated, widespread use of biomarker assays and multimodal preventive therapies will face regulatory, logistical, and compliance hurdles. Cost, insurance coverage, and patient adherence must all be addressed before integration into standard practice.

Taken together, these limitations underscore that while the framework is conceptually promising, significant clinical research and infrastructure development will be required to translate it into practice. The emerging evidence from *Nature* (2025) is encouraging, as it validates the core premise that lithium deficiency itself may be pathogenic, supporting our hypothesis while also emphasizing the need for careful clinical validation.

## Conclusion

Neurodegenerative diseases such as Alzheimer’s disease (AD) and Parkinson’s disease (PD) are complex conditions driven by disruptions in homeostasis, chronic inflammation, and oxidative stress. Our integrated framework emphasizes the early detection of disease through biomarkers, the central role of oxidative stress in disease progression, and the potential of multimodal therapeutic strategies, including antioxidants, lithium, and transcranial magnetic stimulation (TMS), to prevent or slow neurodegeneration.

A core premise of this model is that early detection of oxidative stress and inflammation, combined with timely therapeutic interventions, may alter the trajectory of these diseases. The addition of viral contributions (HSV-1, EBV) further highlights the interplay between infection, oxidative damage, and neurodegeneration, underscoring the need for novel preventive approaches.

We also recognize important limitations, including the variability of antioxidant trials, accessibility of TMS, and the risks of lithium at higher doses. However, the recent *Nature* study (Aron et al., 2025) offers compelling validation of our model: lithium deficiency was shown to accelerate amyloid and tau pathology, microglial activation, and cognitive decline in AD mouse models, while lithium supplementation prevented these effects. These findings provide a powerful proof-of-concept that supports our preventive framework while also highlighting the urgent need to explore optimal dosing and safety in humans.

By framing neurodegeneration as a preventable process rather than an inevitable outcome, our model shifts the focus from symptomatic management to proactive intervention. While obstacles remain, the integration of molecular mechanisms, validated biomarkers, and multimodal therapies, now reinforced by emerging preclinical evidence, offers both scientific promise and translational potential for the future of prevention in neurodegenerative disease.

## Data Availability

The original contributions presented in the study are included in the article/supplementary material, further inquiries can be directed to the corresponding author/s.
