# Correction: The interplay of homeostasis, inflammation, and oxidative stress in neurodegenerative disorders: the role of biological markers, antioxidants, lithium, and TMS — a proposed framework for preventing neurodegenerative disorders through biomarkers and multimodal therapies

**DOI:** 10.3389/fnagi.2026.2009736

**Published:** 2026-10-28

**Authors:** Leonard Lado, Aruna Misir

**Affiliations:** 1 Lado Healing Institute, Bonita Springs, FL, United States; 2 Xavier University School of Medicine, Oranjestad, Netherlands

**Keywords:** neurodegenarative disease, oxidative stress, biomarkers, lithium, transcranial magenetic stimulation

Following publication, errors were identified in the reference list of the published article. The errors comprised references with incorrect bibliographic details or DOIs pointing to unrelated papers, and references that could not be located in indexed databases. All identified errors are corrected below.

In the published article, in the **Reference** section, (Butterfield and Halliwell, 2019) was erroneously written as “Butterfield, D. A. and Halliwell, B. (2019). Oxidative stress and Alzheimer's disease: a continuing story. Neurobiol. Aging 85, 40–50. doi: 10.1016/j.neurobiolaging.2019.06.003”. It should be “Butterfield, D. A., and Halliwell, B. (2019). Oxidative stress, dysfunctional glucose metabolism and Alzheimer's disease. *Nat. Rev. Neurosci*. 20, 148–160. doi: 10.1038/s41583-019-0132-6”.

In the published article, in the **Reference** section, (Chapleau et al., 2022) was erroneously written as “Chapleau, M., Iaccarino, L., Soleimani-Meigooni, D., and Rabinovici, G. D. (2022). PET imaging in Alzheimer's disease: insights and clinical applications. J. Nucl. Med. 63, 13S−19S. doi: 10.2967/jnumed.121.263195”. It should be “Chapleau, M., Iaccarino, L., Soleimani-Meigooni, D., and Rabinovici, G. D. (2022). The role of amyloid PET in imaging neurodegenerative disorders: a review. *J. Nucl. Med*. 63(Suppl. 1), 13S−19S. doi: 10.2967/jnumed.121.263195”.

In the published article, in the **Reference** section, (Engel et al., 2006) was erroneously written as “Engel, T, Hernández, F, Avila, J, and Lucas JJ (2006). Full reversal of Alzheimer's disease-like phenotype in a mouse model with conditional overexpression of GSK-3β by lithium treatment. Neurochem. 99, 683–690. doi: 10.1111/j.1471-4159.2006.04108.x”. It should be “Engel, T., Hernández, F., Avila, J., and Lucas, J. J. (2006). Full reversal of Alzheimer's disease-like phenotype in a mouse model with conditional overexpression of glycogen synthase kinase-3. *J. Neurosci*. 26, 5083–5090. doi: 10.1523/JNEUROSCI.0604-06.2006”.

In the published article, in the **Reference** section, (Itzhaki et al., 2016) was erroneously written as “Itzhaki, R. F., Lathe, R., Balin, B. J., Ball, M. J., Bearer, E. L., Braak, H., et al. (2016). Herpes simplex virus type I and Alzheimer's disease: increasing evidence for a major role of the virus. Front. Aging Neurosci. 8:12. doi: 10.3389/fnagi.2016.00012”. It should be “Itzhaki, R. F., Lathe, R., Balin, B. J., Ball, M. J., Bearer, E. L., Braak, H., et al. (2016). Microbes and Alzheimer's disease. *J. Alzheimers Dis*. 51, 979–984. doi: 10.3233/JAD-160152”.

In the published article, in the **Reference** section, (Rabey et al., 2013) was erroneously written as “Rabey, J. M., Dobronevsky, E., Aichenbaum, S., Gonen, O., and Marton, R. (2013). Repetitive TMS in Alzheimer's. Journal of Neuropsychiatry and Clinical Neurosciences 25, 123–129. doi: 10.1176/appi.neuropsych.12030055”. It should be “Rabey, J. M., Dobronevsky, E., Aichenbaum, S., Gonen, O., Marton, R. G., and Khaigrekht, M. (2013). Repetitive transcranial magnetic stimulation combined with cognitive training is a safe and effective modality for the treatment of Alzheimer's disease: a randomized, double-blind study. *J. Neural Transm*. 120, 813–819. doi: 10.1007/s00702-012-0902-z”.

In the published article, in the **Reference** section, (Sbodio et al., 2019) was erroneously written as “Sbodio, J. I., Snyder, S. H., and Paul, B. D. (2018). Redox mechanisms in neurodegeneration: from disease to therapy. Journal of Neurochemistry 42, 823–827. doi: 10.1016/j.tins.2019.07.004”. It should be “Sbodio, J. I., Snyder, S. H., and Paul, B. D. (2019). Redox mechanisms in neurodegeneration: from disease outcomes to therapeutic opportunities. *Antioxid. Redox Signal*. 30, 1450–1499. doi: 10.1089/ars.2017.7321”.

In the published article, in the **Reference** section, (Subramaniam and Chesselet, 2013) was erroneously written as “Subramaniam, S. R. and Chesselet, M. F. (2013). Mitochondrial dysfunction and oxidative stress in Parkinson's disease. Prog. Neurobiol. 106-107, 17–32. doi: 10.1016/j.pneurobio.2013.04.002”. It should be “Subramaniam, S. R., and Chesselet, M. F. (2013). Mitochondrial dysfunction and oxidative stress in Parkinson's disease. *Prog. Neurobiol*. 106–107, 17–32. doi: 10.1016/j.pneurobio.2013.04.004”.

In the published article, in the **Reference** section, (Bjelakovic et al., 2014) was erroneously written as “Bjelakovic, G., Nikolova, D., and Gluud, C. (2014). Antioxidant supplements for prevention of mortality in healthy participants and patients with various diseases. Cochrane Database Syst. Rev. 2012:CD007176. doi: 10.1002/14651858.CD007176.pub2”. It should be “Bjelakovic, G., Nikolova, D., Gluud, L. L., Simonetti, R. G., and Gluud, C. (2012). Antioxidant supplements for prevention of mortality in healthy participants and patients with various diseases. *Cochrane Database Syst. Rev*. 2012:CD007176. doi: 10.1002/14651858.CD007176.pub2”.

In the published article, in the **Reference** section, (Bashir et al., 2022) was erroneously written as “Bashir S, Ahmed N, Khan T, Ali R, Akram M, Farooq U. Role of transcranial magnetic stimulation in modifying brain pathology in Alzheimer's disease. Front Neurol 13:908563. doi: 10.3389/fneur.2022.908563”. It should be “Bashir, S., Uzair, M., Abualait, T., Arshad, M., Khallaf, R. A., Niaz, A., et al. (2022). Effects of transcranial magnetic stimulation on neurobiological changes in Alzheimer's disease (review). *Mol. Med. Rep*. 25:109. doi: 10.3892/mmr.2022.12625”.

In the published article, in the **Reference** section, (Velioglu et al., 2021) was erroneously written as “Velioglu HA, Ozdemir RA, Bagci A, Erturk IE, Cebi M, Gur G, et al. Left parietal rTMS improves memory and reduces oxidative stress in patients with Alzheimer's disease: a randomized, sham-controlled trial. J Neural Transm 128:365–375. doi: 10.1007/s00702-020-02300-5”. It should be “Velioglu, H. A., Hanoglu, L., Bayraktaroglu, Z., Toprak, G., Guler, E. M., Bektay, M. Y. et al. (2021). Left lateral parietal rTMS improves cognition and modulates resting brain connectivity in patients with Alzheimer's disease: possible role of BDNF and oxidative stress. *Neurobiol. Learn. Mem*. 180:107410. doi: 10.1016/j.nlm.2021.107410”.

In the published article, in the Reference section, (Wang et al., 2024) was erroneously written as “Wang, M., Zhou, S., Yang, H., Lopez, P., and Zhao, L. (2024). Repetitive transcranial magnetic stimulation improves cognition, depression, and walking ability in patients with Parkinson's disease. *BMC Neurol*. 24 [epub ahead of print]. doi: 10.1186/s12883-024-03791-7”. It should be “Wang, M., Zhang, W., and Zang, W. (2024). Repetitive transcranial magnetic stimulation improves cognition, depression, and walking ability in patients with Parkinson's disease: a meta-analysis. *BMC Neurol*. 24:490. doi: 10.1186/s12883-024-03990-9”.

In the published article, the citation (Iqbal and Grundke-Iqbal, 2005), listed in the **Reference** section as “Iqbal, K. and Grundke-Iqbal, I. (2005). Alzheimer's neurofibrillary pathology: therapeutic targets and hopes for the future. J. Alzheimers Dis. 7:243–248. doi: 10.3233/JAD-2005-7307” could not be verified against any indexed source and has been replaced. The in-text citations in the Figure 1 caption and in section **Therapeutic approaches**, subsection *Limitations and challenges*, paragraph 5 have been updated as follows:

“Lithium inhibits glycogen synthase kinase-3β (GSK-3β), a key enzyme implicated in tau phosphorylation (Engel et al., 2006; Forlenza et al., 2014).”

“Many are elevated in multiple neurological and systemic conditions, and assay variability remains a challenge (Forlenza et al., 2014).”

In the published article, the citation (Smith and Jones, 2022), listed in the **Reference** section as “Smith J, Jones M (2022). Role of GFAP in brain inflammation and oxidative stress. Neuroinflammation Rev 14:112–125” could not be verified against any indexed source and has been replaced. The in-text citation in section **The role of homeostasis in inflammation and oxidative stress**, subsection *Biochemical markers of body and brain*, paragraph 1, has been updated as follows:

“Relevance: GFAP is a cytoskeletal protein expressed in astrocytes, reflecting astrocytic activation in response to stress or injury. Elevated GFAP indicates blood–brain barrier (BBB) disruption, oxidative stress, and glial reactivity (Pelkmans et al., 2024).”

In the published article, the citation (Martin et al., 2021), listed in the **Reference** section as “Martin L, Foster J, Garcia M, Silva C (2021). S100B as a marker in Alzheimer's disease. Alzheimers Biomarkers 12:321–328” could not be verified against any indexed source and has been replaced. The in-text citation in section **The role of homeostasis in inflammation and oxidative stress**, subsection *S100B – marker of blood–brain barrier disruption and astrocytic activation*, paragraph 1, has been updated as follows:

“Relevance: S100B is a calcium-binding protein secreted by astrocytes. Elevated levels indicate BBB disruption, oxidative stress, and astrocytic reactivity (Janigro et al., 2022).”

In the published article, the citation (Johnson et al., 2022), listed in the **Reference** section as “Johnson P, Wright E, Berman L, Klein A (2022). NfL in Alzheimer's pathology: a critical analysis. Neurodegener Dis 22:145–155” could not be verified against any indexed source and has been replaced. The in-text citations in section **The role of homeostasis in inflammation and oxidative stress**, subsection *Neurofilament light chain – marker of neuronal injury*, paragraph 2, has been updated as follows:

“In AD: NfL elevations correlate with tau pathology, synaptic loss, and cognitive decline (Jin et al., 2019). Longitudinal studies demonstrate that higher NfL levels predict faster cortical atrophy and conversion from mild cognitive impairment to Alzheimer's disease (Villa et al., 2020; Jin et al., 2019). This makes NfL both a diagnostic biomarker and a tool for tracking disease trajectory.”

In the published article, the citation (Brown et al., 2020), listed in the **Reference** section as “Brown K, Singh A, Tan L, Zhang Y, Clark R, Hughes P (2020). Oxidative stress and astrocytic activation in Parkinson's disease. Parkinsonism Relat Disord 75:45–53” could not be verified against any indexed source and has been replaced. The in-text citation in section **The role of homeostasis in inflammation and oxidative stress**, subsection *Biochemical markers of body and brain*, paragraph 3, has been updated as follows:

“In PD: GFAP levels rise in the substantia nigra, a region with high oxidative load. Chronic astrocytic activation in PD suggests insufficient compensation against oxidative stress and inflammation (Schulz et al., 2021).”

In the published article, the citation (Black et al., 2022), listed in the **Reference** section as “Black S, Miller J, Roberts C, Patel V, Green D (2022). Glial activation and YKL-40 in Parkinson's disease. Parkinsons Mov Disord 37:1425–1434” could not be verified against any indexed source. The in-text citation in section in section **The role of homeostasis in inflammation and oxidative stress**, subsection *YKL-40 (CHI3L1) – marker of neuroinflammation and tissue remodeling*, paragraph 3, has been updated as follows:

“In PD: YKL-40 increases in regions of glial activation and dopaminergic loss, reflecting the persistent inflammatory milieu in PD (Schulz et al., 2021).”

In the published article, the citation (Lopez et al., 2023), listed in the **Reference** section as “Lopez P, Kim J, Hernandez A, Ruiz L, Alvarez T (2023). Astrocytic markers and BBB dysfunction in PD. J Neurol Disord 11:201–209” could not be verified against any indexed source and has been replaced. The in-text citation in section **The role of homeostasis in inflammation and oxidative stress**, subsection *YKL-40 (CHI3L1) – marker of neuroinflammation and tissue remodeling*, paragraph 4, has been updated as follows:

“By stabilizing glial activity, lithium may lower YKL-40 levels and restore homeostasis, although this has not yet been tested directly.”

In the published article, the citation (Davis et al., 2022), listed in the **Reference** section as “Davis K, Morgan A, Chu R, Lee C, Adams T (2022). Lithium and blood-brain barrier protection. Neuroprotective Strateg J 9:115–127” could not be verified against any indexed source and has been replaced. The in-text citation in section **The role of homeostasis in inflammation and oxidative stress**, subsection *S100B – marker of blood–brain barrier disruption and astrocytic activation*, paragraph 4, has been updated as follows:

“Evidence suggests lithium lowers S100B levels by attenuating astrocytic overactivation, thereby contributing to neuroprotection (Nassar and Azab, 2014).”

In the published article, the citation (Chen Y et al., 2020), listed in the **Reference** section as “Chen Y, Zhao P, Wu Y, He L, Zhang J, Wang L (2020). YKL-40 levels in Alzheimer's disease: correlations with severity. Alzheimers Dis J 5:201–212” could not be verified against any indexed source and has been replaced. The in-text citation in section **The role of homeostasis in inflammation and oxidative stress**, subsection *YKL-40 (CHI3L1) – marker of neuroinflammation and tissue remodeling*, paragraph 2 has been updated as follows:

“In AD: Elevated YKL-40 levels correlate with amyloid plaque burden and neuroinflammation, tracking with cognitive decline and disease severity (Nordengen et al., 2019).”

In the published article, the citation (Lee et al., 2021), listed in the **Reference** section as “Lee H, Chen R, Zhang Y, Li T, Chen Q (2021). Astrocyte activation in Alzheimer's disease: mechanisms and biomarkers. Alzheimers Dement 17:1073–1085. doi: 10.1002/alz.12295” contained an incorrect DOI that resolved to a different article and the cited authors and title did not correspond to any indexed source. This reference has been replaced, and the in-text citation in section **The role of homeostasis in inflammation and oxidative stress**, subsection *Biochemical markers of body and brain*, paragraph 2 has been updated as follows:

“In AD: Increased GFAP is observed around amyloid plaques and correlates with astrocytic activation, oxidative burden, and disease severity (Pelkmans et al., 2024).”

In the published article, **Therapeutic approaches**, paragraph 3, the citation for (Bjelakovic et al., 2012) was incorrectly written as (Bjelakovic et al., 2014). The sentence now reads: “N-Acetylcysteine (NAC): A precursor to glutathione, NAC replenishes intracellular antioxidant defenses and mitigates oxidative damage (Bjelakovic et al., 2012).”

In the published article, **Therapeutic approaches**, subsection *Limitations and challenges*, paragraph 2, the citation for (Bjelakovic et al., 2012) was incorrectly written as (Bjelakovic et al., 2014). The sentence now reads: “Variability of antioxidant trials: Clinical studies of antioxidant compounds have shown inconsistent outcomes, with some demonstrating neuroprotective effects and others reporting little benefit (Bjelakovic et al., 2012).”

In the published article, the citation (Serrano et al., 2024), listed in the **Reference** section as “Serrano I, Lopez A, Wang M, Chen Z, Yu Q (2024). Clinical and neurophysiological effects of rTMS in PD. J Neuroeng Rehabil 2024:11” could not be verified against any indexed source and has been removed from the reference list.

In the section **Therapeutic approaches**, subsection *Synergistic potential of TMS and antioxidants*, paragraph 1, the corresponding in-text citation has been removed. The sentence now reads: “Clinical studies also support this interaction: in Parkinson's disease, rTMS improved cognition, mood, and walking ability (Wang et al., 2024), benefits that could be potentiated in the presence of optimized redox balance.”

Similarly, in the section **Therapeutic approaches**, subsection *Synergistic potential of TMS and antioxidants*, paragraph 4, the corresponding in-text citation has been removed. The sentence now reads: “While current studies focus primarily on motor and cognitive symptoms, future trials integrating antioxidants could yield groundbreaking insights into disease modification.”

In the section **Therapeutic approaches**, subsection *Role of transcranial magnetic stimulation in Alzheimer's disease*, paragraph 3, the study by Velioglu et al. was inadvertently referred to as a 2022 study. The correct publication year is 2021.

The sentence has been corrected to: “In their 2021 study, Velioglu et al. explored the therapeutic effects of 20 Hz repetitive transcranial magnetic stimulation (rTMS) applied to the left lateral parietal cortex in patients with Alzheimer's disease (AD).”

In the published article, Section Lithium in Parkinson's and Alzheimer's diseases, the following sentence: “Moreover, lithium has been shown to decrease beta-amyloid production by modulating secretase activity, reducing plaque formation [91].”

Should be corrected to: “Moreover, lithium has been shown to decrease beta-amyloid production by modulating secretase activity, reducing plaque formation (Phiel et al., 2003).”

In the published article, the reference “Carter, T., Alvarez, M., Hsu, L., Reynolds, K., and Wong, J. (2020). Inflammation and sTREM2 as biomarkers in Alzheimer's disease. *Alzheimer's Res Ther* 12:56. doi: 10.1186/s13195-020-00605-0” was erroneously included in the Reference section. The correct reference should be “Ma, L. Z., Tan, L., Bi, Y. L., et al. (2020). Dynamic changes of CSF sTREM2 in preclinical Alzheimer's disease: the CABLE study. *Mol. Neurodegener*. 15:25. doi: 10.1186/s13024-020-00374-8”.

In the published article, the reference “Patel, V., Harrison, J., Thomas, E., and Li, K. (2022). sTREM2 levels in Parkinson's disease. *Neurosci. Lett*. 771:136405. doi: 10.1016/j.neulet.2021.136405” was erroneously included in the Reference section. The correct reference should be “Peng, G., Qiu, J., Liu, H., et al. (2019). Analysis of cerebrospinal fluid soluble TREM2 and polymorphisms in sporadic Parkinson's disease in a Chinese population. *J. Mol. Neurosci*. 70, 294–301. doi: 10.1007/s12031-019-01424-7”.

In the published article, the reference “Wang, H., and Zhou, Y. (2021). Microglial activation and sTREM2 in neuroinflammation. *J. Immunol*. 206, 1123–1132. doi: 10.4049/jimmunol.2001234” was erroneously included in the Reference section. The correct reference should be “Nordengen, K., Kirsebom, B. E., Henjum, K., et al. (2019). Glial activation and inflammation along the Alzheimer's disease continuum. *J. Neuroinflammation* 16:46. doi: 10.1186/s12974-019-1399-2”.

In the published article, the reference “White, R., Anderson, P., Lee, C., and Huang, M. (2021). YKL-40 in neuroinflammation and glial activation. *J. Neuroinflammation* 18:102. doi: 10.1186/s12974-021-02117-5” was erroneously included in the Reference section. The correct reference should be “Pelkmans, W., Shekari, M., Brugulat-Serrat, A., et al. (2024). Astrocyte biomarkers GFAP and YKL-40 mediate early Alzheimer's disease progression. *Alzheimers Dement*. 20, 483–493. doi: 10.1002/alz.13450”.

In the published article, the reference “Taylor, S., Richards, M., Kim, H., and Wong, C. (2022). Neuroprotective effects of lithium on axonal damage. *Neurotherapeutics* 19, 786–795. doi: 10.1007/s13311-022-01192-8” was erroneously included in the Reference section. The correct reference should be “Wang, M., Zhang, W., and Zang, W. (2024). Repetitive transcranial magnetic stimulation improves cognition, depression, and walking ability in patients with Parkinson's disease: a meta-analysis. *BMC Neurol*. 24:490. doi: 10.1186/s12883-024-03990-9”.

In the published article, the reference “Lin, Q., Zhou, Y., Wang, S., Li, H., Liu, X., and Chen, Z. (2023). Lithium's anti-inflammatory properties: evidence from clinical studies. *Front. Neurol*. 14:1152003. doi: 10.3389/fneur.2023.1152003” was erroneously included in the Reference section. This reference should be removed from the reference list.

In the published article, the reference “Wen, Y., and Li, W. (2022). Neuroprotective effects of methylene blue in models of neurodegenerative diseases. *Neurotherapeutics* 19, 13–23. doi: 10.1007/s13311-021-01107-9”. The correct reference should be “Tucker, D., Lu, Y., and Zhang, Q. (2018). From mitochondrial function to neuroprotection, an emerging role for methylene blue. *Mol. Neurobiol*. 55, 5137–5153. doi: 10.1007/s12035-017-0712-2”.

In the published article, the reference “B8. Zhao, L., Cheng, M., Xu, Y., Li, Y., and Ren, B. (2023). Lithium and astrocyte modulation in neurodegenerative disorders. Neuropharmacology 222:109330. doi: 10.1016/j.neuropharm.2023.109330” should be removed. In the original article, in section “**Biochemical markers of body and brain**” the sentence “Lithium may attenuate astrocytic stress and lower GFAP levels by stabilizing inflammatory responses and promoting neurogenesis. Preclinical studies suggest lithium modulates astrocytic activity, potentially reducing BBB disruption and glial reactivity (Zhao et al., 2023).” should be changed to “Lithium may attenuate astrocytic stress and lower GFAP levels by stabilizing inflammatory responses and promoting neurogenesis, although this has not yet been demonstrated directly.”

In the published article, in the section **The role of homeostasis in inflammation and oxidative stress**, subsection *Chronic inflammation and neurodegeneration*, the sentence “This chronic inflammatory state is a major contributor to the progression of neurodegenerative diseases, acting in concert with oxidative stress and other pathological mechanisms (Nathan and Ding, 2010; Biswas et al., 2016).” should read “This chronic inflammatory state is a major contributor to the progression of neurodegenerative diseases, acting in concert with oxidative stress and other pathological mechanisms (Nathan and Ding, 2010; Rock et al., 2010; Biswas et al., 2016).”

The original version of this article has been updated.

## References

[B1] Jin M. Cao L. Dai Y. P. (2019). Role of neurofilament light chain as a potential biomarker for Alzheimer's disease: a correlative meta-analysis. Front. Aging Neurosci. 11:254. doi: 10.3389/fnagi.2019.00254 31572170 PMC6753203

[B2] Schulz I. Kruse N. Gera R. G. Kremer T. Cedarbaum J. Barbour R. Zago W. Schade S. Otte B. Bartl M. Hutten S. J. Trenkwalder C. Mollenhauer B. (2021). Systematic assessment of 10 biomarker candidates focusing on α-synuclein-related disorders. Mov. Disord. 36, 2874–2887. doi: 10.1002/mds.28738 34363416

